# Epigenetic remodelling of enhancers in response to estrogen deprivation and re-stimulation

**DOI:** 10.1093/nar/gkab697

**Published:** 2021-08-17

**Authors:** Athena Sklias, Andrea Halaburkova, Ludovica Vanzan, Nora Fernandez Jimenez, Cyrille Cuenin, Liacine Bouaoun, Vincent Cahais, Victor Ythier, Aurélie Sallé, Claire Renard, Geoffroy Durand, Florence Le Calvez-Kelm, Rita Khoueiry, Rabih Murr, Zdenko Herceg

**Affiliations:** Epigenetics Group, International Agency for Research on Cancer (IARC), 69372 Lyon Cedex 08, France; Epigenetics Group, International Agency for Research on Cancer (IARC), 69372 Lyon Cedex 08, France; Department of Genetic Medicine and Development (GEDEV), University of Geneva, Geneva, Switzerland; Department of Genetics, Physical Anthropology and Animal Physiology, University of the Basque Country (UPV/EHU), Biocruces-Bizkaia Health Research Institute, Leioa, Basque Country 48940, Spain; Epigenetics Group, International Agency for Research on Cancer (IARC), 69372 Lyon Cedex 08, France; Section of Environment and Radiation, International Agency for Research on Cancer (IARC), 69372 Lyon Cedex 08, France; Epigenetics Group, International Agency for Research on Cancer (IARC), 69372 Lyon Cedex 08, France; Department of Genetic Medicine and Development (GEDEV), University of Geneva, Geneva, Switzerland; Epigenetics Group, International Agency for Research on Cancer (IARC), 69372 Lyon Cedex 08, France; Epigenetics Group, International Agency for Research on Cancer (IARC), 69372 Lyon Cedex 08, France; Genetic Cancer Susceptibility Group, International Agency for Research on Cancer (IARC), Lyon, France; Genetic Cancer Susceptibility Group, International Agency for Research on Cancer (IARC), Lyon, France; Epigenetics Group, International Agency for Research on Cancer (IARC), 69372 Lyon Cedex 08, France; Department of Genetic Medicine and Development (GEDEV), University of Geneva, Geneva, Switzerland; Institute for Genetics and Genomics in Geneva (iGE3), University of Geneva, Geneva, Switzerland; Epigenetics Group, International Agency for Research on Cancer (IARC), 69372 Lyon Cedex 08, France

## Abstract

Estrogen hormones are implicated in a majority of breast cancers and estrogen receptor alpha (ER), the main nuclear factor mediating estrogen signaling, orchestrates a complex molecular circuitry that is not yet fully elucidated. Here, we investigated genome-wide DNA methylation, histone acetylation and transcription after estradiol (E2) deprivation and re-stimulation to better characterize the ability of ER to coordinate gene regulation. We found that E2 deprivation mostly resulted in DNA hypermethylation and histone deacetylation in enhancers. Transcriptome analysis revealed that E2 deprivation leads to a global down-regulation in gene expression, and more specifically of TET2 demethylase that may be involved in the DNA hypermethylation following short-term E2 deprivation. Further enrichment analysis of transcription factor (TF) binding and motif occurrence highlights the importance of ER connection mainly with two partner TF families, AP-1 and FOX. These interactions take place in the proximity of E2 deprivation-mediated differentially methylated and histone acetylated enhancers. Finally, while most deprivation-dependent epigenetic changes were reversed following E2 re-stimulation, DNA hypermethylation and H3K27 deacetylation at certain enhancers were partially retained. Overall, these results show that inactivation of ER mediates rapid and mostly reversible epigenetic changes at enhancers, and bring new insight into early events, which may ultimately lead to endocrine resistance.

## INTRODUCTION

Despite the continuous efforts for prevention and surveillance, breast cancer (BC) remains the most common cancer in women across the world (1). Among established risk factors, steroid hormones, notably estrogens, have been recognized as key players in BC, and current chemopreventive strategies target hormonally responsive breast tumours. BCs are classified into different molecular subtypes mainly according to the presence of estrogen receptor (ER), progesterone receptor (PR) and epidermal growth factor receptor 2 (HER2), and their expression tends to determine the treatment approach. While on average only 7% of normal mammary epithelial cells express ER, more than 70% of breast tumours are ER-positive (ER+) which suggests that ER+ cells are more prone to oncogenesis, presumably due to their ability to respond to a variety of biological (endogenous estrogens) and environmental stimuli (such as steroid-like molecules) (2,3). In addition, treatment of ER+ BC patients is based on this characteristic as it lends itself readily to anti-estrogen therapy that down-regulates ER signalling and hence inhibits ER-induced cell proliferation. Unfortunately, patients regularly develop a non-reversible resistance to anti-estrogen therapy, underscoring the importance of understanding ER pathway regulation in hormone-driven breast cancer (4).

The central role of ER in BC development, progression and treatment has prompted the development of mechanistic models of the activity of this nuclear factor to study the regulation of transcription, chromatin structure and epigenetic marks. The nuclear receptor super-family to which ER belongs, has the dual characteristic of acting both as a TF binding to DNA and as a ligand receptor binding to a variety of steroid-like molecules, including estrogens, androgens or progestogens (5). To decipher the molecular mechanisms that ER triggers upon ligand activation, estrogen-responding breast cancer cell lines, often MCF-7 that is widely used by ENCODE (6), are deprived of steroid stimuli for several days and re-stimulated with agonist E2, after which various readouts can be measured. Following ligand binding, ER can either up-regulate or down-regulate gene expression, which is achieved by recruiting different protein complexes (7). For instance, ER’s interaction with epigenetic regulatory factors (ERFs) TET2, p300 or MLL3 and with TFs such as FOXA1 or GATA3, is associated with positive gene regulation while inversely, recruitment of polycomb repressive complex 2 (PRC2) and DNMTs results in transcriptional repression (8–12). Furthermore, interactions with a variety of cofactors allow ER to establish chromatin interactions both in *cis* and in *trans*, mediating concerted gene expression by bringing promoters and enhancers in close proximity in three-dimensional space (13,14). Recent studies performed in cell culture models and patient samples have shown that changes in high-order assemblies of transcription factors, including GATA3 and AP-1, can reorganize the landscape of ERα-bound enhancers, resulting in gene program transitions that promote cancer cell plasticity and development of therapy resistance (15). Enhancers are usually predicted by the presence of H3K4me1, TF footprints, and p300 binding, while enhancer activity is determined through H3K27 acetylation and enhancer RNA (eRNA) transcription (16,17).

In an effort to better characterize enhancers, several studies have placed their attention on the DNA methylation (DNAm) profiles of these regulatory elements and they showed that the low and intermediate DNAm levels measured at these locations are most likely the result of dynamic ERF and TF binding (18–20). It is now well admitted that the majority of ER binding occurs on enhancers and it was recently shown that ER binding on enhancers requires the presence of the methylcytosine dioxygenase TET2 (8,12). The integration of tumour and cell line data showed that higher ER expression and binding correlates with lower levels of DNAm in a specific panel of enhancers (21–23). However, little is known about the dynamics of ER-dependent epigenetic changes and how variations in DNA methylation are connected with chromatin reconfiguration and changes in genes expression.

In the present study we sought (i) to identify epigenetic changes that are directly attributed to ligand-mediated ER down-regulation, (ii) to investigate the functional impact of these changes, notably in relation to gene transcription, chromatin activity and cooperation with other TFs and (iii) to test whether these changes can be precluded or reversed upon subsequent ER re-activation. By optimizing the E2 deprivation and re-stimulation cell culture protocol that mimic the decrease of E2 levels in blood of breast cancer patients treated with aromatase inhibitors, a treatment that often evolves into an endocrine resistance after prolonged use (24), in conjunction with the latest methylation arrays, transcriptome (RNA-Seq) and chromatin (ChIP-Seq) analyses, we provide new insights into the genome-wide epigenetic dynamics of ligand-mediated ER activity in breast cancer cells. Our results revealed that DNA hypermethylation and histone deacetylation of enhancers is an early response to down-regulation of ER signalling. In addition, TF enrichment analysis revealed the AP-1 transcription factor among the top potent candidate cofactor in mediating ER-specific DNAm maintenance at enhancers. Finally, we observed a partial reversibility of DNAm and histone acetylation changes induced by sequential estrogen deprivation and re-stimulation.

## MATERIALS AND METHODS

### Cell culture and treatments

MCF-7 HTB-22^®^ cells were obtained from the American Type Culture Collection. Prior to E2 deprivation/re-stimulation experiments, MCF-7 were conditioned for two weeks to phenol-red-free DMEM/F-12 supplemented with 10% (v/v) charcoal-stripped fetal bovine serum (csFBS, Sigma-Aldrich), 1% nonessential amino acids, sodium pyruvate and penicillin/streptomycin (Life Technologies) and to a daily addition of 10 nM E2 in 0.1% DMSO (Sigma-Aldrich) at 37°C, in a humidified and 5% CO_2_-enriched atmosphere. For all assays, control cells (CTR) were cultured continuously for 14 days in the above-mentioned conditions, while E2-deprived cells (E2D) were cultured in the same conditions only lacking E2. The re-stimulated cells (ReSt) were E2-deprived for 4 days and re-stimulated with E2 for the 10 following days. Two hours before being harvested, CTR and ReSt cultures were incubated with 10 nM E2 in 0.1% DMSO while E2D were incubated only in 0.1% DMSO. MCF-7 cells that were used as standard controls (STD) as well as in the inhibition and siRNA assays were maintained in the same composition of medium as mentioned above, but with 10% of standard FBS (Eurobio).

### ICI 182 780 inhibition

**Figure 1.**
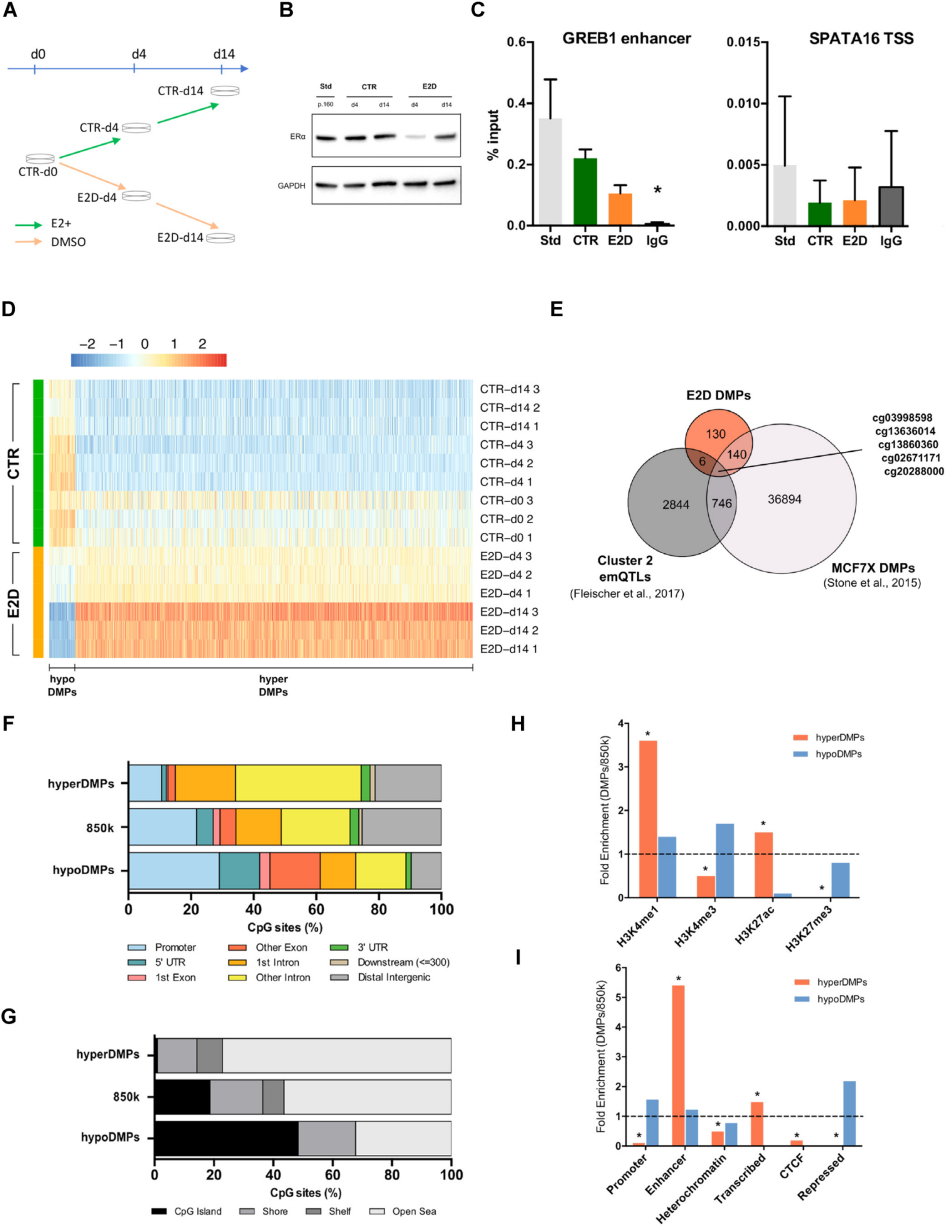
E2 deprivation leads to time-increasing hypermethylation of CpGs in enhancers. (**A**) MCF-7 cultured in control conditions (CTR, charcoal-stripped serum + 10nm of E2) were deprived from E2 for 4 and 14 days (E2D). All experiments were performed in triplicates. (**B**) ERα protein levels in MCF-7 cells grown in standard medium till passage 160 (Std; phenol-red and complete FBS), in CTR and E2D. GAPDH was used as an internal control. (**C**) ER binding levels in GREB1 enhancer (left) and in SPATA16 promoter (right) for the same conditions as in (B) at d14 alone, (n = 3). Asterisks indicate significant differences from the IgG (Student's *t*-test, *P*< 0.05). n.s., not significant (Student's *t*-test, *P*> 0.05). (**D**) Heatmap of DMPs between CTR and E2D MCF-7 cells with at least 10% differential methylation (Δβ). (**E**) Overlap of 450k CpG sites between E2D DMPs, estrogen-associated emQTLs in breast tumours (Cluster 2, Fleischer et al., 2017) and long-term E2 deprivation DMPs in MCF7 (MCF7X, Stone et al., 2015, re-analysed with limma, FDR < 0.01; Δβ ≥ 10%). (**F**) Genomic features distribution and (**G**) CpG density of hypermethylated DMPs (hyperDMPs, *n* = 950), all 850k CpG probes (*n* = 866 836) and hypomethylated DMPs (hypoDMPs, *n* = 45). (**H**) Fold-enrichment of DMPs overlapping with publicly available histone ChIP-seq data and (**I**) ChromHMM annotations originated from MCF-7 cells (Hnisz, 2013). Asterisks (*) mark significant enrichments (*P*< 10e–5, Fisher test). Distributions and enrichments are shown for DMPs with ≥10% Δβ.

For the inhibition assay, MCF-7 cells were continuously cultured in presence of 1 μM of ER antagonist ICI 182 780 (ICI) and CTR cells were cultured in 10% FBS medium containing 0.1% DMSO. To study the persistence/reversibility of the effect, cells were cultured with ICI for 4 days then placed in the same medium as used for the control for 10 days to re-activate ER activity (ReAc). For both deprivation and inhibition assays, cells were cultured and collected in triplicates at day 0, 4 and 14 (Figures 1A, 5A and Supplementary Figure S1E). The effectiveness of ICI treatment was checked by measuring the expression levels of ER-target gene *GREB1* (Supplementary Figure S1F).

### siRNA

MCF-7 at 30% confluence were transfected separetaly with 10 nM of *ESR1*, *TET2* or non-targeting siRNA and 1 nM of FOS siRNAs (Dharmacon, On-Target Plus siRNA) using 2.5 μl of RNAiMAX lipofectamine for a 6-well format (Life Technologies) as recommended by the manufacturer. Cells were maintained in the lipofection for 48 h and then replaced by fresh medium. Cells were harvested 120h from the day of transfection. RNA and DNA were extracted as mentioned in DNA methylation analysis. Expression of ESR1 and GREB1 transcripts and proteins was measured to verify the efficiency of silencing (Supplementary Figure S1B and C). The percentage of knock-down efficiency was calculated as the average of (100 – (2^–ΔCt_siRNA_ – 2–ΔCt_siNT_)) × 100.

### DNA methylation analysis

For all assays, total DNA was extracted using AllPrep DNA/RNA Mini kit (Qiagen) and was quantified by Qubit dsDNA HS kit (Invitrogen). For the genome-wide methylation analysis of CTR, E2D and ReSt samples, DNA was bisulfite converted using the EZ DNA Methylation kit (Zymo Research) and 250 ng were hybridized on an Infinium MethylationEPIC array, referred to as 850k from this point (Illumina). Processing and normalization of data followed by identification of differentially methylated positions (DMPs) was performed as described earlier (25) with a detection *P*-value ≥0.01. To determine the effect of E2D deprivation on DNAm, we used a linear regression model where we compared E2D (*n* = 6) treatment to CTR (*n* = 9) while adjusting for timepoint and applying a differential methylation cutoff of 10% (false discovery rate, FDR < 0.05; Δβ > 10%). Two variables were taken into account for the linear regression used to define the DMPs: treatment (CTR versus E2D) and time. To exclude the possibility that DMPs are obtained by chance, we shuffled the treatment annotations 10 times and we ran the linear regression again. Not a single DMP came up when treatment groups were mixed. Then, to test the reversibility of the impact of E2 deprivation, we applied a linear regression model between CTR and ReSt samples at d14. *Minfi*, *limma*, *FDb.InfiniumMethylation.hg19* and *ChIPseeker* R/Bioconductor packages were used for obtaining annotating the DMPs (26,27). Analysis of DNAm in validation and in inhibition assays at a single CpG level was done by pyrosequencing, as previously described (28). The effect of treatment for each timepoint was tested with pairwise comparisons using a Mann-Whitney test. Primer sequences used for pyrosequencing may be found in Supplementary Table S1.

### Oxidative bisulfite treatment and hydroxymethylated DNA immunoprecipitation

Oxidative bisulfite treatment (oxBS) of 1 μg of DNA was performed with TrueMethyl oxBS Module (Nugen), as described in (29). Levels of 5-hydroxymethylcytosine (5hmC) were measured by pyrosequencing and they were calculated by subtracting the proportion of Cs obtained in oxBS from BS (5hmC% = 5mC%_BS_ – 5mC%_oxBS_). For hydroxymethylated DNA immunoprecipitation (hMeDIP) total DNA was extracted using the Gentra Puregene kit (Qiagen), 1.25 μg of which were sonicated for 6 cycles [15 s ON/90 s OFF] on a Bioruptor Pico. 5hmC was immunoprecipitated using 2.5 μg of anti-5hmC antibody as per the manufacturer's instructions (Auto hMeDIP Kit, Diagenode). The enrichment was measured using qPCR that was performed as previously described in (28) following these conditions: denaturation 95°C 5min, amplification [95°C 15 s, 60°C 60 s, 72°C 60 s] × 40 cycles, melting curve 95°C 60 s, 55–90°C with 0.5°C increment every 5 s. The percentage of precipitated 5hmC over input was calculated as such as the result of 2^[(Ct(_10%input_) – 3.32) – Ct(_hmetDNA-IP_)] × 100%.

### Chromatin immunoprecipitation

MCF-7 cells were cultured in CTR, E2D and ReSt conditions as mentioned above and were fixed with 1% formaldehyde at d14 for 8 and 10 minutes for histone marks and TFs respectively. One million cells were sonicated and processed with the iDeal ChIP-seq kit for histones and 4 million cells were used to immunoprecipitate TFs using the iDeal ChIP-qPCR kit as per the manufacturer's protocol (Diagenode). Chromatin was sonicated over 14 cycles of [30 s ON/30 s OFF] on a Bioruptor Pico and was immunoprecipitated with 2.5 μg of anti-H3K27ac, anti-H3K4me1 and anti-H3K27me3 antibodies (respectively, ab4729, ab8895, ab6002, Abcam) in duplicates and with 2.5 μg of anti-ERalpha antibody (C15100066 Diagenode) in triplicates. H3K27ac ChIP and input libraries were prepared using the TruSeq ChIP Sample Prep kit and we performed paired-end sequencing with the NextSeq 500/550 High Output Kit v2.5 (150 Cycles) (Illumina). Fastq files were trimmed using *TrimGalore* for adapters and a minimum quality of *Q* > 30 (Babraham Bioinformatics), then mapped on hg19 using BWA and further processed as previously described (9,30). Mapped read numbers ranged between 125 and 165 million. Peaks were then called with MACS2 (2.1.1.20160309) against input and were filtered *ad hoc* for <1.5 kb width, *q* < 0.01 and fold-change from input >5 (31). For downstream analysis we worked with irreproducible-discovery-rate (IDR) thresholded peaks. Differentially bound (DB) regions were identified with *DiffBind* between different groups at d14 and only DB peaks with |log_2_FC| >1 were considered (32). ChIP-qPCR was performed in the same conditions as for hMeDIP (see previous section). To test ER binding, a GREB1 enhancer (chr2:11638671–11638726) was used as a positive control. The promoter of SPATA16 (chr3:172859020–172859121), a gene expressed only in testis, was used as negative control. The percentage of input was calculated as such as the result of 2^[(Ct_(1% input)_ – 6.64) – Ct_(IP sample)_] × 100. Primer sequences used for validation are listed in Supplementary Table S2.

### Western blot

Western blots were performed by loading 25–30 μg of total MCF-7 proteins on 4–15% precast polyacrylamide gels (Bio-Rad). The blots were incubated overnight at +4°C with the following specific primary antibodies: anti-ERalpha (1:2000, C15100066 Diagenode), anti-FOS and anti-TET2 (1:750, ab222699 and 1:1000 ab94580 Abcam) and anti-GAPDH (1:2000, SC-32233 Santa-Cruz) for loading control. These were followed by incubation with species-matched secondary antibodies. The detection of the antibody hybridization was done with the Clarity Western ECL mix (Bio-Rad).

### Gene expression analysis

Total RNA was extracted along with DNA samples using the AllPrep DNA/RNA Mini kit (Qiagen). One μg of RNA was used for TruSeq RNA library preparation (Illumina) following the manufacturer's protocol and single-end sequencing was performed on HiSeq 2500 yielding at least 20M 50 bp-reads for each sample. Following quality control, reads were trimmed at 30 bp and mapped on hg19 with hisat2 with mapping efficiencies of ≥87% (33). Read counts were generated from bam files using htseq-count function and genes with ≤10 counts-per-million in at least two samples were eliminated (34). Differentially expressed genes (DEGs) were identified between CTR and E2D adjusting for time and between CTR and ReSt at d14 using *edgeR* (FDR < 0.05, |log_2_FC| > 1) (35). Targeted expression analysis of specific genes was performed by quantitative RT-PCR as previously described (28). Briefly 500 ng of RNA were retro-transcribed using MMLV-RT and oligo(dT) primers (Invitrogen). Target regions were amplified from 2.5 ng of cDNA using SYBR Green (Biorad) and relative expression was determined using RPLP0 as a housekeeping gene. The PCR conditions used were as follows: denaturation 95°C 5min, amplification [95°C 15 s, 61°C 30 s] × 40 cycles, melting curve 95°C 60 s, 55–90°C with 0.5°C increment every 5 s. The expression was measured in technical and biological triplicates and the signal was normalized over the housekeeping gene *RPLP0*. The global effect of treatment was tested with pairwise comparisons using a Mann–Whitney test (R v3.5.1). Primer sequences are listed in Supplementary Table S3.

### *De novo* DNA motif analysis, TF and histone enrichment and statistics

The ChEA database, provided online by the EnrichR collection of tools, was used for a first evaluation of the enrichment of TF binding on DEGs and genes mapping close to DMPs and DB H3K27ac regions (36). Only TF enrichments with an adjusted *P*-value <0.05 were considered. A *de novo* TF motif enrichment analysis was performed after enlarging the genomic window to 250 bp around DMPs and to 1 kb for DB H3K27ac, using the *findMotifsGenome.pl* function from HOMER v.4.8.3 (37). Whole genomic ranges were kept as such for *de novo* motif search in publicly available TF ChIP peaks. For overlaps with specific TFs, histone marks and ChromHMM annotations, we downloaded published histone and TF ChIP-seq peaks (Supplementary Table S4) and we overlapped them with the genomic ranges of DMPs, DB H3K27ac peaks and DEGs produced in this study. All public datasets used were based on MCF-7 cells cultured in E2 containing media. E2-dependent peaks were derived from ER peaks that were present in E2-treated MCF-7 and absent from E2 deprived cells (38). The enrichment was calculated as the ratio of genomic-range overlap of the differential hits over the overlap with the total CpG probes, H3K27ac peaks or expressed genes respectively for each dataset (differential/expected). More precisely, for DNAm we used the genomic ranges of all probes on the 850k array, for H3K27ac marks we used the IDR-thresholded peaks of CTR at d14 and for transcription we used the genomic range of expressed genes. Fisher's exact test (*P* < 10^–5^) was used to evaluate the significance of enrichments.

### Pathway enrichment analysis

A gene set enrichment analysis was performed on differentially expressed genes revealed by RNA-seq using collection H from MSigDb (v7.1) (39). For non-coding regions (DMPs and H3K27ac peak) GREAT annotations was used.

### Visualization

For the visualization of H3K27ac peaks on IGV (2.4.19), bam files were normalized using the reads per genome coverage (RPGC) normalization method. BigWig or bedgraph format was used to visualize publicly available datasets. Graphpad 6.0 was used for bar and scatter plots, R 3.5.1 was used for volcano and box plots, NMF package (0.21.0) was used for heatmaps and Inkscape 0.92 was used to assemble layouts.

## RESULTS

### E2 deprivation leads to time-dependent hypermethylation of CpGs in enhancers

To investigate the effect of inactivation of a steroid nuclear receptor on DNAm patterns across time, the ER-positive cell line MCF-7 was first deprived of E2, *stricto sensu*, as outlined in Figure 1A. In previous studies on ER activity and endocrine resistance, ER-positive cells were usually cultured in medium containing charcoal-stripped serum (csFBS), a serum that has been stripped of the large majority of its lipophilic substrates, including E2, androgens, growth factors and cytokines (22,40) and compared to control counterparts cultured in medium containing standard serum. Therefore, in those studies the effect of E2 was confounded with a panel of other molecules. To address this limitation, we generated cell culture conditions where control (CTR) and E2-deprived (E2D) ER-positive MCF-7 cells were conditioned to the same background: phenol-red free medium with csFBS. While E2D cells were deprived for up to 14 days, CTR cells were supplemented daily with E2 which allows to analyse the effects strictly related to E2 deprivation (Figure 1A). We evaluated ER levels in our different culture conditions and observed that ER protein levels in CTRs were comparable to culture conditions with standard FBS (STD) (Figure 1B), demonstrating the equivalence of our model to previous studies focusing on E2-modulated ER. Moreover, even though the transcription levels of *ESR1* mRNA fluctuated slightly (Supplementary Figure S1A), we observed a drop of ER protein levels at 4 and 14 days of E2 deprivation (Figure 1B), confirming that our deprivation conditions modulate ER and possibly its downstream targets. In line with that, we observed a decrease of ER binding on a known binding site within GREB1 enhancer, a well-described ER target (Figure 1C) (41). In deprived conditions, GREB1 also showed a decrease in expression in a similar manner to when ESR1 expression was reduced by siRNA knockdown or after inhibition of the ER pathway by the common ER antagonist drug ICI (Supplementary Figure S1B–F). Taken together, these results indicate that our protocol modulates ER signalling according to the known ER pathway and is valid for analysing the impact of such modulation on cells.

Following the validation of the impact of the deprivation protocol on ER pathway, we aimed to assess the changes in DNA methylation induced by this modulation. To this end, DNA extracted from the three times points (day 0, 4 and 14; d0, d4 and d14 respectively) were processed on an 850k array that measures DNAm levels across more than 850,000 CpGs (that includes over 300 000 CpGs located in enhancer regions). Due to a potential effect of the time in culture on DNAm, the log-transformed methylation values were fitted in a linear regression model contrasting for treatment and adjusting for time (Supplementary Figure S2). Therefore, considering together two timepoints (d4 and d14) of E2-deprivation, we identified 995 DMPs (Δβ > 10%, FDR < 0.05) between CTR and E2D samples (Figure 1D). Among those prominent DMPs, 95% were hypermethylated (*n* = 950, hyperDMPs) while the remaining were hypomethylated (*n* = 45, hypoDMPs), and the magnitude of these changes increased in time (Figure 1D, Δβ[CTR-d4 versus E2D-d4] < Δβ[CTR-d14 versus E2D-d14]). In addition, we observed a difference in DNAm levels between CTR groups at d4 and d14 and the initial group at d0 that could be due to cell culture. However, DNA methylation variations are probably not due to changes in cell cycle and accumulation of cells in a particular phase of the mitosis as we did not observe a change in cell proliferation following E2 deprivation. The gain of methylation was further validated at a subset of top E2-dependent DMPs by pyrosequencing in two independent E2 deprivation experiments as well as by siRNA knock-down of ESR1 (Supplementary Figure S3A and B). These results show that E2 deprivation leads to a significant increase of DNAm as early as 4 days after the beginning of deprivation and this wide ranging hypermethylation becomes more pronounced with time.

Among the 995 identified DMPs, about a third (*n* = 281) can also be found on the former version of the Illumina array that covers over 450,000 CpG sites (450k). Therefore, to further validate and identify specific differences in DNAm in response to E2 deprivation, we overlapped the 450k DMPs that were obtained following our short-term (d4 and d14) E2-deprivation with two published sets of 450k DMPs: the first set of CpG sites from estrogen-associated emQTLs that are differentially methylated between ER-positive and ER-negative breast tumours (Cluster 2, (21)) and a second set of DMPs obtained by a long-term E2 deprivation of MCF7 (MCF7X, (22)). Interestingly 145 DMPs were common between cells deprived from E2 for a short (E2D) and long period suggesting that those events represent an early response of cells to E2 deprivation. Five CpGs were common among all the three datasets, four of which (cg03998598, cg13860360, cg02671171, cg20288000) were hypermethylated in ER-negative tumours as well as in short and long-term E2 deprived MCF-7 cells (Figure 1E). In particular, cg20288000 site is found in an enhancer of CASZ1, a gene coding for a TF that is involved in different cancers, including epithelial ovarian cancer (42) consistent with the notion that hypermethylation of enhancers may be observed after reduced ER activity in tumours.

Analysis of genomic features exhibiting differential methylation in response to E2 deprivation showed that hypermethylated DMPs (hyperDMPs) were markedly enriched in intronic and CpG-poor contexts (Figure 1F and G). In contrast, hypoDMPs, represented by a low number of DMPs, were not significantly enriched in any particular feature. In order to further investigate the potential function of these DNAm changes, we overlapped DMPs with publicly available ChIP-Seq datasets for histone modifications that are commonly used to define chromatin state and that were available in MCF-7 cultured in standard medium containing E2 among other hormones (Supplementary Table S4). Our analysis revealed that hyperDMPs were positively enriched in both H3K4me1 and H3K27ac marks (79.0% and 27.9% of hyperDMPs, respectively), which suggests that hypermethylation occurs in putative active enhancer regions (Figure 1H). In contrast, we observed that hyperDMPs were depleted (14.2%) in H3K4me3 marks (known to overlap with promoter regions), while no hyperDMPs were found in active H3K27me3 regions (repressive mark) (Figure 1H). To further support the specific occurrence of these changes in regulatory regions, we overlapped the DMPs with ChromHMM annotations of predicted chromatin states in MCF-7 (43). We confirmed that the hyperDMPs were significantly represented in enhancer and transcribed regions (49.8% and 14.9%, respectively), whereas they were lowly represented in promoter, CTCF-bound or repressed regions (1.9%, 0.8% and 0.0%, respectively) (Figure 1I). These results indicate that the increase in DNAm in response to E2 deprivation mostly occurs within intronic regions that contain enhancers.

### E2 deprivation globally leads to a decrease of active histone marks at enhancers

**Figure 2.**
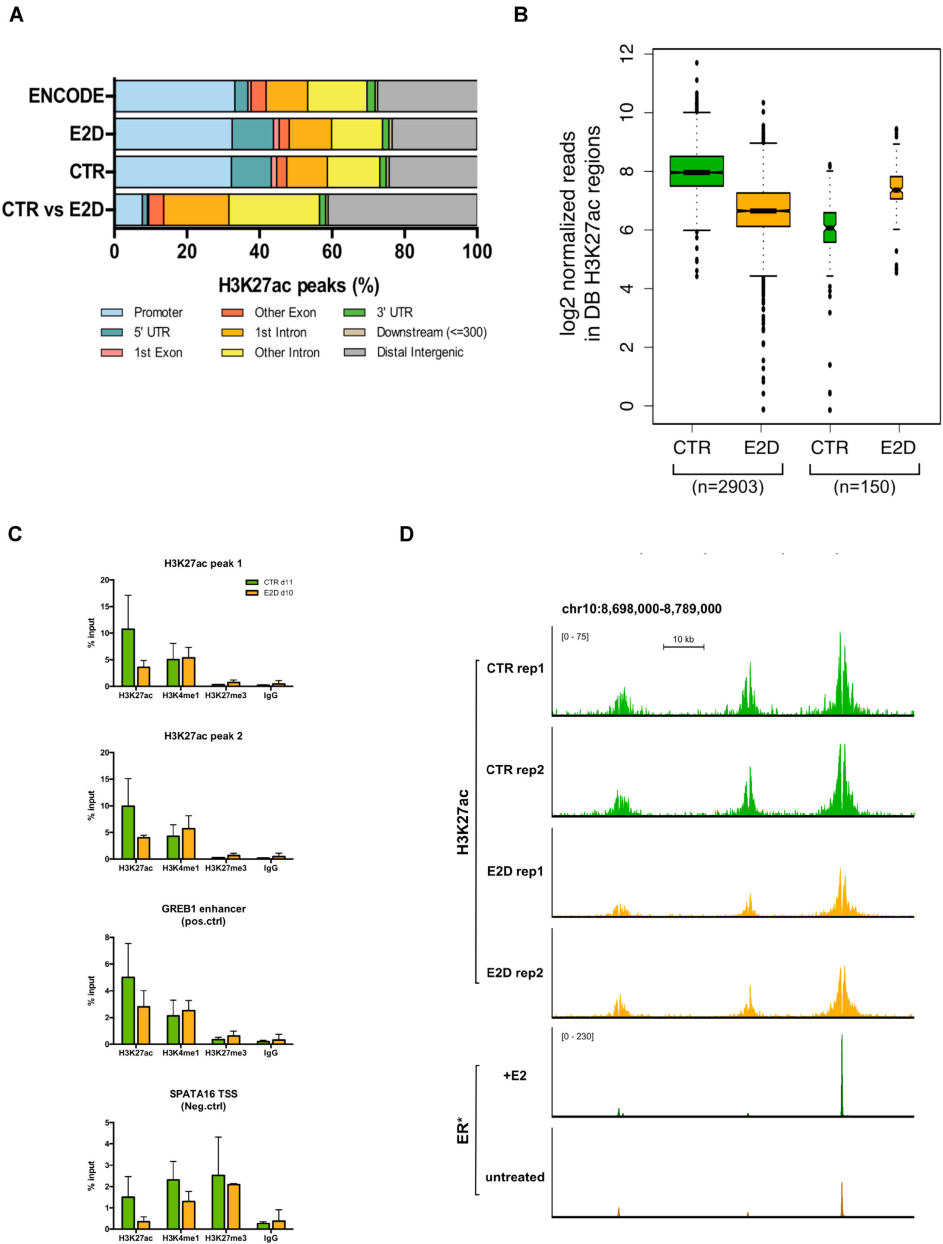
E2 deprivation globally leads to a loss of H3K27ac at enhancers. (**A**) Genomic features distribution of ENCODE H3K27ac peaks (n = 33631), H3K27ac IDR thresholded peaks for CTR and E2D at d14 (*n* = 31 090 and *n* = 32 995) as well as for the differentially bound peaks between the two groups (DB H3K27ac, *n* = 3053, |log_2_FC| > 1, FDR < 0.05, *n* = 2). (**B**) Global decrease of histone acetylation expressed in log_2_ normalized reads in DB H3K27ac regions in CTR and E2D (|lo_g2_FC| > 1, FDR < 0.05). DB peaks were separated in decreasing and in increasing acetylation in response to E2 deprivation. (**C**) H3K27ac, H3K4me1, H3K27me3 and IgG binding levels after 11 days of CTR and E2D treatments. qPCR data was normalized over input DNA and presented as the average ± SD of duplicates. Groups were compared using a Student's *t*-test within each histone mark. (**D**) Genome browser snapshot of H3K27ac normalized ChIP-seq reads in CTR and E2D conditions and ER ChIP-seq reads in E2-treated and untreated MCF-7 (*GSE72249, Swinstead *et al.* 2016) upstream of GATA3 gene (chr10:8 698 000–8 789 000).

Because the hyperDMPs were found to strongly overlap with enhancer elements, we sought to evaluate enhancer activity by measuring changes in H3K27ac levels, which distinguishes active from primed, poised and inactive enhancers (44). For this, we performed ChIP-seq to identify regions enriched for H3K27ac in CTR and E2D groups at d14. Altogether, H3K27ac peaks were evenly detected across promoter, intronic and intergenic regions (Figure 2A). However, the 3053 differentially enriched (DB) regions (|log_2_FC| >1, FDR < 0.05) obtained by the comparison of CTR and E2D groups, were highly enriched and located in a similar genomic context to DMPs, that is, in intronic and intergenic regions (42.9% and 41.1%, respectively) that are mostly corresponding to enhancers (Figure 2A and Supplementary Figure S4A) (43). The large majority of H3K27ac DB regions showed a lower signal in E2D cells compared to CTR cells (Figure 2B and C). Independently of the treatment, H3K27me3 marks were found to be low at H3K27ac sites (Figure 2C and Supplementary Figure S4B). While DMPs and DB regions rarely co-localized (5.0% of DMPs in a 10 kb window of a DB H3K27ac peak), a significant number of genes exhibited both changes within a 1 Mb distance (Supplementary Figure S4C), suggesting a coordinated but distant regulation of enhancers in *cis* that depends on the presence of E2. Furthermore, under E2-stimulated conditions, 25.9% of DB peaks co-localized with ER binding events and almost half of the latter (11.2% of total DB peaks) overlapped with E2-specific ER peaks (38) (Figure 2D, Supplementary Figure S4D). Taken together these results point to an inactivation of enhancers accompanied by decreased active H3K27ac mark in response to E2 deprivation, which is consistent with a hypermethylation of DMPs and potential impact on gene transcription.

### Down-regulation of TET2 expression following E2 deprivation and hypermethylation of enhancers

**Figure 3.**
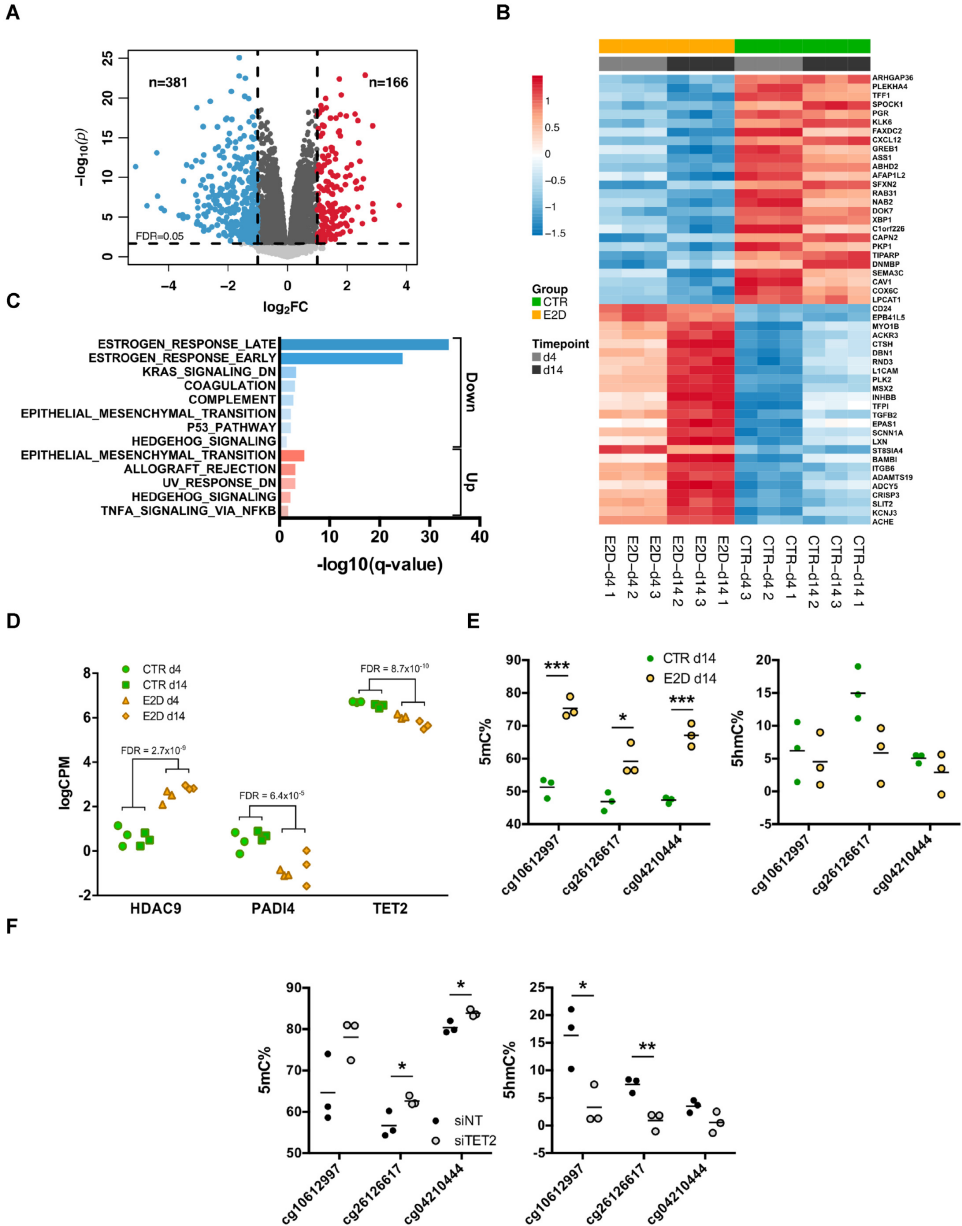
Down-regulation of TET2 expression following E2 deprivation and hypermethylation of enhancers. (**A**) Distribution of –log_10_(*P*-values) of differentially expressed genes (DEGs) according to log_2_ fold-change of expression. Coloured dots represent down- and up-regulated DEGs with an absolute log_2_(FC)>1 (blue and red) and a FDR < 0.05 (dashed horizontal). DEGs that were differentially expressed with an absolute log_2_(FC)<1 are coloured in dark grey. (**B**) Top 50 differentially expressed genes (FDR < 0.05) between CTR and E2D at d4 and d14. (**C**) Gene set enrichment analysis of down-regulated and up-regulated DEGs (MSigDB, database H, hypergeometric test). (**D**) Expression of epigenetic remodeling factors HDAC9, PADI4 and TET2 in response to E2 deprivation shown as log of counts per million mapped reads (CPM) (FDR < 0.05). (**E**) 5′-methylcytosine (left) and 5′-hydroxymethylcytosine (right) levels of top DMPs in CTR and E2D groups at d14 (Student's t-test, * *P* < 0.05, ** *P* < 0.01, *** *P* < 0.001) and (**F**) 5′-methylcytosine (left) and 5′-hydroxymethylcytosine (right) levels of top DMPs in control (siNT) and TET2 silenced (siTET2) cells (Student's *t*-test, * *P* < 0.05, ** *P* < 0.01, *** *P* < 0.001).

To further characterize the genome-wide impact of ER activity on gene regulation and how this is related to epigenetic status of enhancers, we performed genome-wide transcriptome analysis by NGS-sequencing (RNA-Seq) in CTR and E2D conditions, in the same samples used for the methylome assays. The analysis revealed that the deprivation of E2 led to 547 differentially expressed genes (DEGs) (|log_2_FC| >1 and FDR < 0.05), from which 71.1% were down-regulated (Figure 3A and B). The down-regulated fraction was particularly enriched in genes involved in estrogen-related response (Figure 3C). Canonical ER targets were found, as expected, among down-regulated genes, such as GREB1, TFF1 and PGR (Figure 3B). The global downregulation in gene expression is in line with the observed general increase of DNAm and loss of histone acetylation marks. Nevertheless, the overlap between DEGs and genes containing DMPs or DB H3K27ac peaks in *cis* was relatively low compared to the total number of genes (Supplementary Figure S5A). 40 out of the 547 DEGs overlapped with genes located nearby a DMP and 110 DEGs were associated with DB H3K27ac peaks (Supplementary Figure S5A) indicating that the enhancers may not systematically regulate the expression of the nearest genes.

To identify epigenetic regulators that may be responsible for those DNAm and histone acetylation changes, we examined ERFs that could be differentially expressed in response to E2 deprivation. For this, we searched for gene expression changes among 426 previously identified ERFs (45) and found that HDAC9, PADI4 and TET2 were significantly deregulated (FDR < 0.05), the latter having the highest expression levels (Figure 3D). The expression of these three ERFs changed significantly and similarly to E2D after 4 and 14 days of treatment with ICI further validating the impact of ER pathway deregulation on their expression (Supplementary Figure S5B). Because E2 deprivation affected the expression of the 5mC demethylase TET2 and not the other members of the ten-eleven translocation (TET) family (TET1 and TET3, Supplementary Figure S5C), we investigated the role of TET2 in DNA methylation changes induced by E2 deprivation. TET2 knockdown (KD) revealed a decrease in ER targets (such as *GREB1)*, suggesting a putative role of the TET2 downregulation (observed in E2D) in loss of expression of ER targets (Supplementary Figure S5D and E). We next measured 5-hydroxymethylcytosine (5hmC) levels, which reflect the activity of TET enzymes, in a selection of top E2-responsive DMPs. Consistent with the array data, 5mC levels increased after 14 days of E2-deprivation, whereas 5hmC levels were noticeably decreased (Figure 3E). A similar trend was also observed when hMeDIP-qPCR experiments were performed (Supplementary Figure S5F). Measuring 5mC and 5hmC levels on the same sites following TET2 KD further revealed an increase of 5mC levels, similar albeit less pronounced as the E2D treatment, and to a significantly stronger decrease of 5hmC levels (Figure 3F). Together, the decrease of ER activity and downregulation of TET2 expression is associated with an increase in 5mC levels and decrease in 5hmC levels at DMPs of enhancers.

### Identifying key ER cofactors in E2-dependent transcriptional regulation

**Figure 4.**
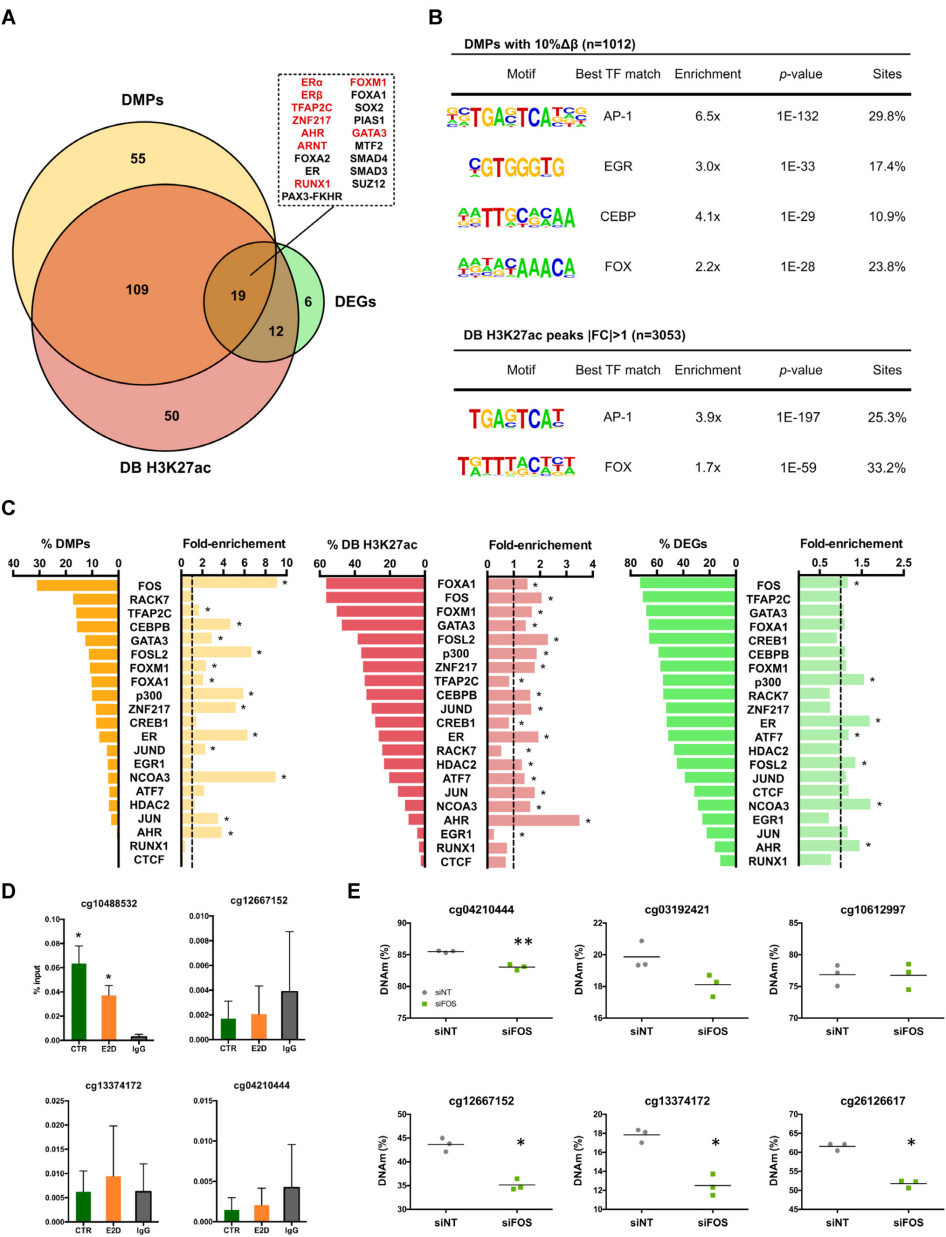
Key cofactors in E2-mediated chromatin and gene regulation. (**A**) Enrichment of TF binding over the nearest genes to DMPs (n = 829 genes), the nearest genes to differentially bound H3K27ac marks (*n* = 577, ≥2 peaks per gene name) and DEGs (*n* = 547). Numbers represent the amount of TFs whose binding was significantly enriched across the different sets of genes (FDR < 0.05). Enrichment of TFs originating from experiments performed in MCF-7 cells are highlighted in red in the upper right panel (ChEA 2016, Kuleshov *et al.* 2016). (**B**) *de novo* motif analysis performed on 14-day-E2-deprivation induced DMPs (top 4 hits shown) and DB H3K27ac (top 2 hits shown). The enrichment is the result of the percentage of the motif occurrence in target sequences over random background genomic sequences. The motif search was expanded over a 500 bp-window for DMPs and a 1 kb-window for DB H3K27ac peaks. (**C**) Overlap of DMPs, DB H3K27ac regions and DEGs with a collection of publicly available TF ChIP-seq datasets in MCF-7. Fold-enrichement was calculated as the fold-change between the percentage of binding overlap within a differential set of hits over the percentage of TF overlap within the total of 850k CpGs, the H3K27ac regions in CTRs at d14 (IDR peaks) and the total fraction of genes that were expressed in the RNA-seq. (**D**) ER binding on a selection of hyperDMPs in CTR and E2D MCF-7 (*n* = 3). IgG is shown only for CTR. Both anti-ER and anti-IgG ChIPs were normalized over input. Asterisks indicate significant differences from the IgG (Student's *t*-test, *P*< 0.05). n.s., not significant (Student's *t*-test, *P*> 0.05) (**E**) 5′-methylcytosine levels of top hyperDMPs 5 days after transfection of siRNA targeting FOS (Student's *t*-test, * *P* < 0.05, ** *P* < 0.01)

Our results show that E2 deprivation leads to DNA hypermethylation and a decrease in histone acetylation of enhancers and that these are partially associated with gene expression down-regulation in *cis* and potentially in *trans*. As it has been shown that the removal of E2 decreases ER binding events and time of residence (38), it is expected that E2 deprivation does not only affect DNA binding of ER but also of its cofactors. We therefore asked whether the regions bearing epigenetic changes could be particularly bound by other TFs. For this we first conducted an enrichment analysis of transcription factor binding sites in the proximity of DMPs, DB H3K27ac and DEGs using the ChEA database (36). Our analysis revealed that in addition to expected enrichment of ERα and ERβ binding events, binding sites of other TFs (including ZNF217, TFAP2C and GATA3) were found enriched in genes that map near DMPs and DB H3K27ac regions (Figure 4A). These findings support previously reported functional and physical protein interactions between these TFs and ER (11,46). Interestingly, the up-regulated fraction of DEGs (*n* = 166) was enriched for binding sites of PRC2 members SUZ12 and EZH2, consistent with the fact that when ER is active, it can also actively repress gene expression (Supplementary Figure S6) (47). TF binding sites within the down-regulated fraction of DEGs—that are active when ER is E2-stimulated—were more enriched in genes containing DMPs. This suggests that common TFs are involved in the maintenance of these genes’ epigenetic marks and their expression.

Although informative, the ChEA database is limited by the number of datasets it contains and provides a broad picture of TF enrichment based on gene names rather than specific genomic coordinates. Therefore, to detect putative DNA-binding TFs in a more unsupervised and focused approach, we searched for ER and other TF motifs in proximity of E2 deprivation-mediated DMPs and DB H3K27ac by performing a *de novo* motif analysis. Our first observation was the lack of any of the motifs corresponding to the above-identified TFs, particularly the ER-binding motif, suggesting that these TFs regulating ER-dependent genes do not bind directly on or in close proximity of DMPs and DB H3K27ac regions. Those observations also suggest that the epigenetically variable enhancers are not necessary directly bound by ER (Figure 4B). Indeed, a low fraction of hyperDMPs and H3K27ac regions (7.3% and 26.0%, respectively) overlapped with ER bound regions detected in MCF-7 cells cultured in presence of E2 (Figure 4C) (38). We validated this trend by ER ChIP-qPCR showing that ER binds only one out of the four selected hyperDMPs (Figure 4D).

Interestingly, we found that the motifs matching AP-1 and FOX TFs, known as principal ER cofactors, were enriched in proximity to both DMPs and DB H3K27ac regions (Figure 4B and C). Based on these findings and the knowledge of reported ER cofactors, we overlapped a set of publicly available TF ChIP-seq datasets in MCF-7 exposed to E2 among other hormones (Supplementary Table S4) with DMPs, DB H3K27ac marks and DEGs identified in this study. We found that FOS and FOSL2 binding, two AP-1 components, were enriched 9.1-fold and 6.7-fold, respectively, across DMPs, and across DB H3K27ac and DEGs (Figure 4C). Although not substantially overlapping, ER, ER cofactor NCOA3 and acetyltranferase p300 that are involved in regulation of transcriptional activity and AHR were significantly enriched across all differential datasets (Figure 4C, Supplementary Figure S7). Moreover, the overlap with the ChEA database confirmed that the promoters mapping closer to DMPs and DB H3K27ac regions can be bound by ER. Inversely, the majority of DB H3K27ac peaks and DEGs overlapped with other known ER cofactors’ binding sites such as FOXA1, GATA3 and ZNF217 but with a less notable enrichment (Figure 4C, middle and right panel). This is in line with the preferential binding of these factors in proximity to active marks or active genes. Together, these findings emphasize the importance of ER tethering and suggest that AP-1 may be directly involved in the maintenance of DNAm. Indeed, downregulation of FOS resulted in loss of DNA methylation at several of CpGs observed to be hypermethylated in E2D (Figure 4E and Supplementary Figure S8A).

As cells’ response to our deprivation protocol might show similarities with the development of resistance in response to anti-estrogen therapy, we reasoned that ER cofactors might play similar roles in the chromatin rearrangements and DNA methylation changes observed in both cases. Therefore, we overlapped JUN (a component of AP-1 complex) ChIP-seq data analysed in MCF-7 cells and MCF-7 cells that acquired resistance following tamoxifen treatment (15) with our DB H3K27ac and DMP sites. Interestingly, we observed a lack of JUN binding at those sites in MCF-7 cells and a gain in binding in ∼25% of lost H3K27ac sites (E2D versus CTR) and ∼10% of hyperDMPs sites (E2D versus CTR) which is suggestive of an important regulatory role of AP-1 complex at those sites (Supplementary Figure S8B).

### Partial reversibility of epigenetic changes following E2 re-stimulation

**Figure 5.**
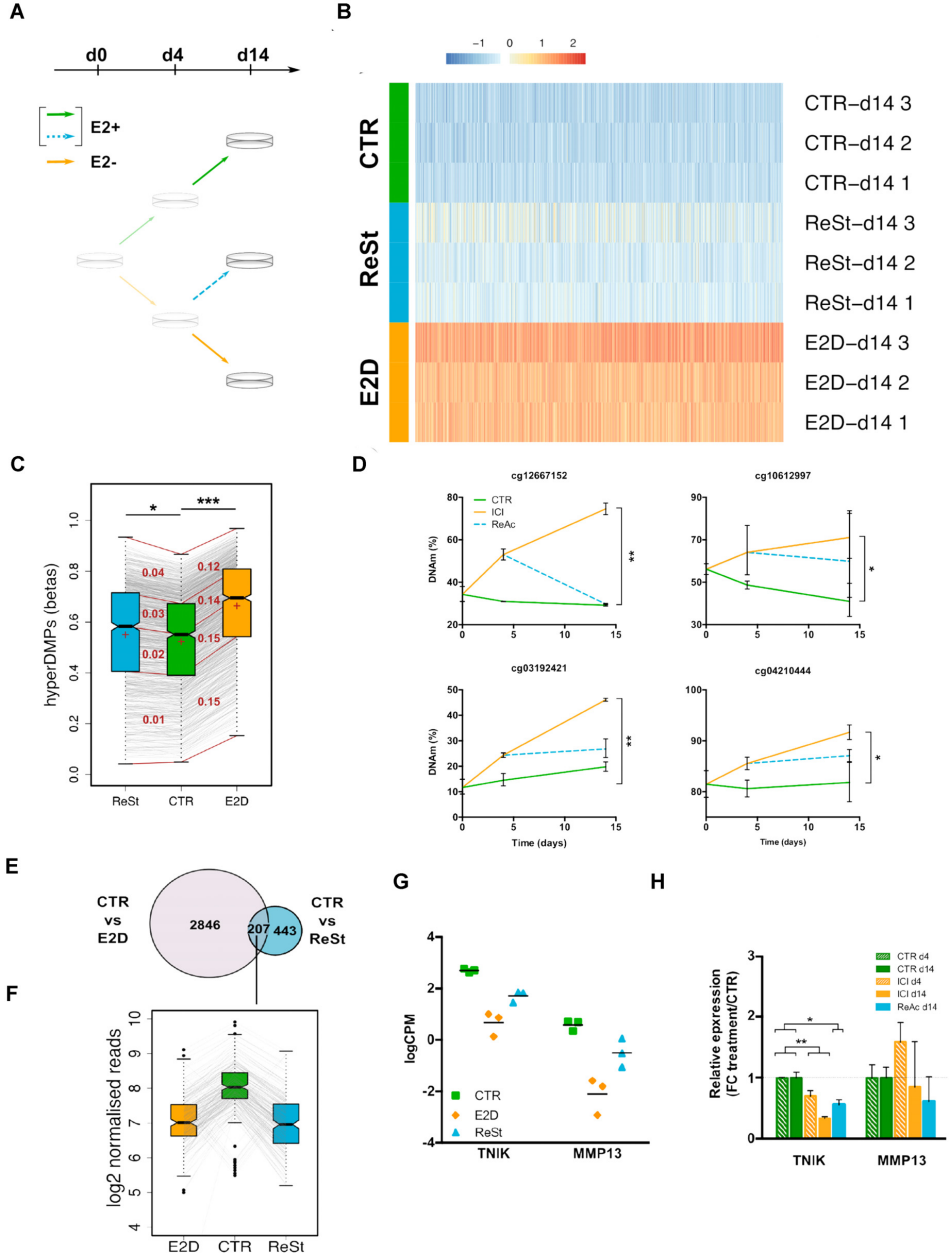
Partial reversibility of epigenetic and transcription changes induced by sequential deactivation and re-activation of ER. (**A**) MCF-7 were deprived of E2 for 4 days, after which they were re-stimulated with E2 for 10 days (ReSt, blue dashed line). (**B**) Heatmap of hypermethylated DMPs (FDR < 0.05; > 10% Δβ) in response to E2-deprivation for CTR, E2D and ReSt at d14. (**C**) Distribution of hypermethylated DMPs in CTR, E2D and ReSt at d14 (CTR versus E2D, n = 950; FDR < 0.05, Δβ > 10%). Box plot: centre lines, median (*Q*_2_); box boundaries, 25% and 75% quartiles (*Q*_1_ and *Q*_3_); top and bottom whiskers, minimum and maximum (*Q*_0_ and *Q*_4_). For each pairwise comparison (ReSt-CTR and CTR-E2D), the quartiles are connected with red lines. In each interquartile range appears in red the mean Δβ between the compared interquartile groups. The mean of each group is shown by a red cross. Asterisks marks significant differences of ReSt and E2D means compared to CTR (Student's *t*-test, * *P* < 0.01 and *** *P* < 10^–5^). (**D**) DNAm levels of top DMPs following DMSO treatment (CTR, green), ICI treatment (ICI, orange), and ICI followed by DMSO treatment (ReAc, blue dashed). Results are shown as the mean of triplicates with 95% of C.I. (Mann–Whitney test at d14, * *P* < 0.05). (**E**) Overlap of differentially bound H3K27ac regions between CTR versus E2D and CTR versus ReSt regions. (**F**) H3K27ac signal in log2 normalized reads of 207 peaks that were significantly different in both CTR versus ReSt and CTR versus E2D comparisons. (**G**) Non reversibility of expression of AP-1 inducer TNIK and AP-1 target gene MMP13 for CTR condition (green square), E2D (orange diamonds) and ReSt (blue triangles) on day 14. Differential expression analysis was performed by contrasting CTR vs ReSt groups among E2-deprivation DEGs, FDR < 0.05, |log_2_FC| > 1. (**H**) gene expression changes following DMSO treatment for 4 and14 days (CTR, green), ICI 182 780 treatment for 14 days (ICI, orange), and 4 days of ICI 182 780 treatment followed by 10 days of DMSO (ReAc, blue dashed). Relative expression is expressed as the fold-change of ICI and ReAc treatment over the CTR of the same timepoint. Results are shown as the mean of triplicates with 95% of C.I. Significance of changes was evaluated with Kruskal–Wallis followed by Dunn's multiple comparison test where crosses indicate overall significance (^+^*P* < 0.05 and ^++^*P* < 0.01) and asterisks indicate pairwise significance (*P < 0.05 and **P < 0.01).

Because epigenetic marks can be dynamic, we reasoned that the changes in DNAm and H3K27ac following E2 deprivation may be reversible when the cells are re-stimulated with E2. To test this, we deprived MCF-7 cells from E2 for 4 days and added back E2 for another 10 days after which we analysed DNAm, H3K27ac and transcription profiles (Figure 5A, Supplementary Figure S9A–C). We first analysed differences in methylation levels between the re-stimulated group (ReSt), the CTRs and E2Ds. We looked for changes across all 850k probes and found no statistically significant differences on global levels of DNAm between individual probes (Figure 5B). Interestingly, when we narrowed the analysis to the previously identified E2D-derived hyperDMPs, the mean DNAm level was significantly higher in the ReSt group in comparison to the CTR group (Figure 5C). It was also notable that hyperDMPs with higher DNAm levels in CTRs (3rd and 4th quartile) had a higher difference with ReSt (Figure 5C). The latter was also observed when targeted methylation analyses was performed at several of the top identified hyperDMPs (Supplementary Figure S9D). The comparison of E2D to ReSt yielded 44 DMPs, all of which were previously detected among the hyperDMPs (CTR vs E2D). To further validate the reversibility of DNAm changes, we used another approach, where we inhibited ER activity with its antagonist, ICI, and measured DNA methylation levels at the top hyperDMPs at day 4 and day 14. To mimic re-stimulation we included also a group where ER was inhibited with ICI for 4 days and reactivated for another 10 days after the removal of ICI (ReAc) (Supplementary Figure S9E). Similar to E2-deprivation, we found that ICI treatment increased DNAm on CpG sites selected among top hyperDMPs (Figure 5D). Although not significantly different, DNAm levels of ReAc group were not equal to CTRs at all tested sites, suggesting partial reversibility of DNAm levels.

We next evaluated changes in chromatin activity after re-stimulation with E2 and found 650 H3K27ac regions that were differentially bound between CTR and ReSt at d14 (Figure 5E). 207 of these regions were also found in the 3053 DB H3K27ac regions that had a lower signal in E2D after comparison with CTR (Figure 5F). The H3K27ac read abundance at these 207 regions was significantly lower in ReSt compared to the CTR group while it was comparable to E2D group, suggesting that H3K27ac levels may have dropped at d4 and remained stable till d14. The majority of regions with lower H3K27ac levels in E2D (*n* = 2846, Figure 5E) recovered their initial levels.

Finally, we evaluated the reversibility of expression levels following E2 re-stimulation by RNAseq analyses and found a recovery of the majority of genes expression following ER re-stimulation (Supplementary Figure S10A). This recovery included the expression of several known ER targets (including CREB1, TFF1 and PGR) as well as ERFs (notably HDAC9, PADI4 and TET2) (Supplementary Figure S10B and C). Similar gene expression recovery was also observed in ReAC cells following ICI treatment (Supplementary Figure S10D). However, we found that only 6 out of 381 E2D-down-regulated DEGs failed to return to the CTR level of expression. Interestingly, two of these 6 more stably altered genes are TNIK and MMP13, respectively an inducer and a target of AP-1 suggesting the possibility of ER regulating AP-1 activity (Figure 5G). In addition, expression of TNIK, but not that of MMP13, decreased with ICI and did not fully recover with the removal of the inhibitor suggesting that AP-1 pathway could also be deregulated on the long run in this setting (Figure 5H).

Globally, these results show that epigenetic changes are largely reversible following E2 re-stimulation. However, a smaller fraction of the observed changes does not recover, even when the expression of certain ERFs does, suggesting the existence of an estrogen-dependent epigenetic memory.

## DISCUSSION

In this study, we combined a cell culture protocol adapted for studying E2 deprivation and restimulation in *stricto sensu* with the latest methylation array that allowed a genome-wide interrogation of methylation states, including a comprehensive panel of enhancers (48). We found that prolonged E2 deprivation and re-stimulation result in time dependent changes in DNAm and histone modifications (histone acetylation) across diverse genomic regions, many of which occur within enhancer elements. This is the first study that comprehensively characterized DNAm and histone acetylation at enhancers in response to a sequential inactivation and re-activation of ER signalling. These observations are consistent with and extend previous studies showing that ligand-mediated ER activity has an impact on the epigenome, through a mechanism that likely involves tethering of ER by other TFs (5,8,21).

While studies have shown that hypermethylation of enhancers may be a long-term consequence of reduced ER activity, our study reveals that gain of methylation at enhancers occurs shortly following ER downregulation (induced either by E2 deprivation or ER inhibition) and that this hypermethylation increases in time. Previous studies have established that DNA hypermethylation at gene promoters is associated with gene silencing (49). In contrast, there is scant data on the role of DNAm changes at enhancer regions, and the mechanisms that underlie specific targeting of enhancers for hypermethylation is unclear. It is known that specific chromatin interactions, involving DNAm dynamics, are abolished and are replaced by others in response to ER activation and inactivation (13,14,43). Indeed, in some instances DNAm deposition is directed by TF-mediated DNMT recruitment, whereas in others DNAm can be the result of the lack of TF occupancy which exposes these sites to DNMT complexes that have an affinity for unmethylated CpGs (20,50). In 2016, Swinstead *et al.* showed using both ChIPseq and single-molecule tracking that ER binds less frequently and less specifically in the absence of its ligand (38). Based on the above information, ER disengagement from chromatin, induced by E2 deprivation, could make unmethylated CpG sites susceptible to methylation, which is consistent with the observation that methylated DNA represents a default state (51). In an alternative, although not mutually exclusive scenario, E2 deprivation-associated hypermethylation may be the result of decreased demethylating activity at enhancers. Interestingly, we observed both after E2 deprivation and ICI inhibition a decrease in expression of DNA demethylase TET2 (Figure 3D, Supplementary Figure S5B and C). Because the activity of TET2 was associated mainly with gene bodies in a mice model, the enrichment of hyperDMPs in intronic regions could also be the result of TET2 down-regulation (52). Indeed, down-regulation of TET2 in MCF-7 cells resulted in an increase of methylation and decrease of 5hmC at several CpGs similar to what was observed in E2 deprivation conditions. The possibility of a double mechanism explaining hypermethylation is supported by a recent study showing that ER not only recruits TET2 on ER-dependent enhancers, but it also positively regulates TET2 expression in response to E2 induction, and that TET2 knock-out leads to hypermethylation of enhancers (12). This does not exclude an additional possible scenario involving a loss of APOBEC3B (A3B) deaminases at the hyperDMPs. A3B has been proposed to play a role in active DNA demethylation and has also been shown to co-bind with ER on the genome (53). Interestingly, 78 of our detected hyperDMPs overlap with regions previously detected to be co-occupied by A3B and ER in MCF-7 cells treated with E2 (data not shown) (38). These findings suggest that following E2 deprivation the binding of ER and A3B may decrease and their absence at those regions may result in a gain of methylation.

In addition to dynamic levels of DNAm, low density of CpGs and intermediate levels of DNAm are also characteristic of enhancers and have been suggested to play a role in enhancer priming (18,54,55). In line with that, our results revealed that initial DNAm levels at sites that become hypermethylated after E2 deprivation range between 40% and 71% (1st and 3rd quartile) with an average of 55% (Figure 5C). Our ChIP-seq data, showing a decrease of H3K27ac levels after E2 deprivation, are consistent with a decrease of enhancer activity. This finding, together with the absence of overlap with H3K27me3 (Figure 2C and Supplementary Figure S4B), which is characteristic of repressed and poised chromatin states, suggests that the identified enhancers may switch from an active to a primed state in response to E2 deprivation (44,56). ER disengagement from sites carrying DB H3K27ac marks is apparent and correlates with H3K27ac reduction in almost half of the sites that overlap with ER binding (38) (Figure 2D, Supplementary Figure S6). Just like for DNAm and DNMTs, we hypothesize that the reduced binding of ER could open the opportunity to histone deacetylases (HDAC) to act. It is indeed plausible that the decrease of H3K27ac signal could be related to the observed increase of HDAC9 expression, although there is little evidence of a direct relationship between this class IIa HDAC and H3K27ac deacetylation (57,58). Based on these findings we propose that the decreased activity of ER and potentially that of its cofactors renders ER-dependent enhancers prone to DNA hypermethylation and histone deacetylation, ultimately tipping the balance from an active to a primed enhancer configuration (50,59). The decrease of TET2 and the increase of HDAC9 expression that was observed in response to both E2 deprivation and ICI inhibition could contribute to the above mechanism, although further studies are needed to test this hypothesis (Figure 3D, Supplementary Figure S5B and C).

Because the overlap between changes in gene expression and epigenetic marks in *cis* was limited (Supplementary Figure S5A), we focused on the analysis of the TF network of ER based on the assumption that their binding to ER-dependent enhancers may also be affected, and consequently impact the epigenetic landscape. Although, our analysis of enriched binding motifs of TFs (using both a supervised and unsupervised approach) failed to identify ER motifs in proximity to DMPs and DB H3K27ac peaks, whereas ER binding significantly overlapped with DEGs, DMPs and DB H3K27ac regions observed after E2 deprivation (Figure 4). These results may reflect the frequency by which ER regulates genomic regions through a tethering mechanism with other cofactors, rather than through direct binding (Supplementary Table S5) (5,11). For example, the CpG site cg10488532 located 250kb upstream from TET2 TSS is hypermethylated in E2D and is dynamically bound by ER but lacks an ER motif. Interestingly, this CpG is part of a region that is described to be the putative enhancer *E1* of *TET2* and is suggested to be regulated by TET2 itself that may contribute to maintaining active its own enhancer (5,11,12).

Interestingly, AP-1 and FOX motifs were enriched in both DMPs and DB H3K27ac regions, and binding of FOS, an AP-1 component, overlapped with DMPs at significantly more sites than other enriched TFs (9.1× and 31% of DMPs, Figure 4C). The absence of ER motif and the presence of AP-1 motif, combined with a recent study showing that TET2 occupies ER-related enhancers, as well as the results from this study and from others showing that loss of TET2 in MCF-7 leads to hypermethylation at these enhancers (12,52), support the idea that AP-1 might play a bridging role between ER and TET2. In this scenario, AP-1 could be involved in the regulation of local DNAm levels as it was previously shown for FOXA1 (60,61). As FOS silencing led to a hypomethylation of several E2-responsive CpG sites (Figure 4E) and these same CpGs were shown to be hypermethylated when TET2 is lost (Figure 3E and F), it is possible that AP-1 influence TET2 activity at those regions. Similarly, the occurrence of AP-1 motif and the enrichment of acetyltransferase p300 binding sites at DB H3K27ac regions reinforce previously reported AP-1-mediated regulation of histone acetylation by p300 recruitment to enhancers (62–64). Our results suggest an important role of AP-1 at enhancers following cells’ response to changes in the activity of the ER pathway. This is in line with recent studies showing that AP-1 (and other TFs) re-organizes enhancer landscapes resulting in transcriptional transitions that promote tumour phenotypic plasticity and resistance to Tamoxifen (an ER modulator) (65). Although the latter study shows a high recruitment of JUN (a component of AP-1 complex) to gained enhancers in MCF-7 cells that acquired resistance, overlapping JUN binding sites with our DB H3K27ac and DMPs shows a lack of JUN at those sites in MCF-7 cells and a gain in binding in ∼25% of lost H3K27ac sites and ∼10% of hyperDMPs sites. Our results might be reflective of the re-arrangement or recruitment of AP-1 to enhancer regions that occurs at early stages of resistance to therapy. Remarkably, AP-1 and FOX motifs were consistently and highly enriched at all binding sites of all established ER cofactors investigated (namely TFAP2C, p300, ZNF217 and AHR) (Supplementary Figure S7). This is in line with the preferential binding of these factors in proximity to active marks or active genes. FOXA1 and GATA3, followed by FOS binding were also significantly enriched at functional enhancers (defined by an expression–methylation quantitative trait loci analysis) that distinguish ER-positive from ER-negative BC subtypes (21). This raises the possibility that the specific mechanisms are conserved between cell line models and clinical samples.

Our data also showed that the majority of DNA hypermethylation induced by E2 deprivation could be prevented or even reversed when the cells were re-stimulated with E2 after a period of deprivation or after ICI inhibition (Figure 5D). Interestingly, although not significantly different, DNAm levels of the ER-dependent hyperDMPs were higher in the re-stimulated group compared to controls. This trend was more pronounced when DNAm levels were high (>40%) prior deprivation. When CpG sites that had a basal low DNAm (<40%) had almost the same level in ReSt (data not shown). In addition, while the majority of changes in H3K27ac were recovered following E2 re-stimulation, the loss of H3K27ac signal in response to E2 deprivation remained significantly lower in another subset of regions. These results indicate that DNAm and H3K27ac changes are precluded from increasing in some regions while they are reversed in others following ER re-activation, but further experiments are needed to identify their singular features. It was previously shown that tamoxifen treatment, another ER inhibitor, reduces AP-1 binding on ER-dependent sites and is re-located on new binding sites (66). Although the removal of tamoxifen was not tested, re-distribution of AP-1 binding, that is enriched in our DMPs and DB H3K27ac, on other genomic locations could explain the lagging recovery of DNAm and histone acetylation on certain enhancers.

It is noteworthy that at the transcriptional level, TNIK and MMP13 decreased after estrogen deprivation and failed to recover after a prolonged E2 re-stimulation (Figure 5G and H). TNIK is required for JNK1 activation, a kinase that activates the AP-1 complex, whereas MMP13, a metalloprotease, that is regulated through the same signalling pathway, is considered as a marker of breast cancer invasiveness (67,68). This observation combined with the unchanged levels of AP-1 component expression opens the door to the exploration of nongenomic signalling that is not addressed in this study. Although we could not rule out the possibility that duration of deprivation followed by re-stimulation was not sufficiently long enough to observe a full reversal of epigenetic changes, a selective retention of DNA methylation changes, indicative of epigenetic memory (69), is consistent with the notion that the disruption of ER and its impact on ER cofactors’ pathways including AP-1 signalling may be involved in endocrine resistance (66,70,71).

Based on our results, we propose a model in which ligand-activated ER and its cofactors orchestrate an intricate interaction in *cis* and *trans* between promoters and a panel of epigenetically dynamic enhancers (Supplementary Figure S11). Under continuous E2 exposure, AP-1 contributes to the maintenance of balance between TET2 and DNMT proteins in order to keep DNAm at intermediate levels in intronic enhancers while, in collaboration with p300 and FOXA1 proteins, AP-1 preserves histone acetylation in distal enhancers and thereby maintains gene expression. In this scenario, the lagging recovery of DNAm and histone acetylation levels at certain enhancers could be the result of the partial redistribution of AP-1 binding sites away from ER-responsive regions, as it was previously observed in tamoxifen-treated cells (66,70). In parallel to this mechanism, the variation of TET2 and HDAC9 expression levels could contribute to the enhancers’ shift from an active to a primed state.

In summary, this study provides mechanistic insight into the events by which estrogen receptor and its cofactors mediate changes in DNA methylation and chromatin states at enhancers in response to estrogen deprivation and re-stimulation. The selective reversibility and persistence of DNA methylation and histone acetylation changes observed after estrogen deprivation/re-stimulation, suggest a potential mechanism underlying the ‘roots’ of endocrine resistance that commonly develops in response to anti-estrogen therapy. This insight may open new research avenues to investigate epigenetic modifications at enhancers and how estrogen receptor binding may participate in ER positive breast cancer drug/treatment resistance.

## DATA AVAILABILITY

Raw 850k, ChIP-seq and RNA-seq data as well as processed data are deposited on GEO platform (accession number GSE132514).

## Supplementary Material

gkab697_Supplemental_File

## Notes

Present address: Athena Sklias, Center for Integrative Genomics, Génopode Building, Faculty of Biology and Medicine, University of Lausanne, Lausanne, Switzerland.

Present address: Ludovica Vanzan, Institute of Bioengineering, School of Life Sciences, École Polytechnique Fédérale de Lausanne, Lausanne, Switzerland.
