# Supplementary material for: Epigenetic remodelling of enhancers in response to estrogen deprivation and re-stimulation
Source: Nucleic Acids Res. 2021 Aug 17;49(17):9738–54. doi: 10.1093/nar/gkab697 (PMC8464064; doi:10.1093/nar/gkab697)
Supplement: gkab697_Supplemental_File [file gkab697_supplemental_file.pdf]

Supplementary Figures

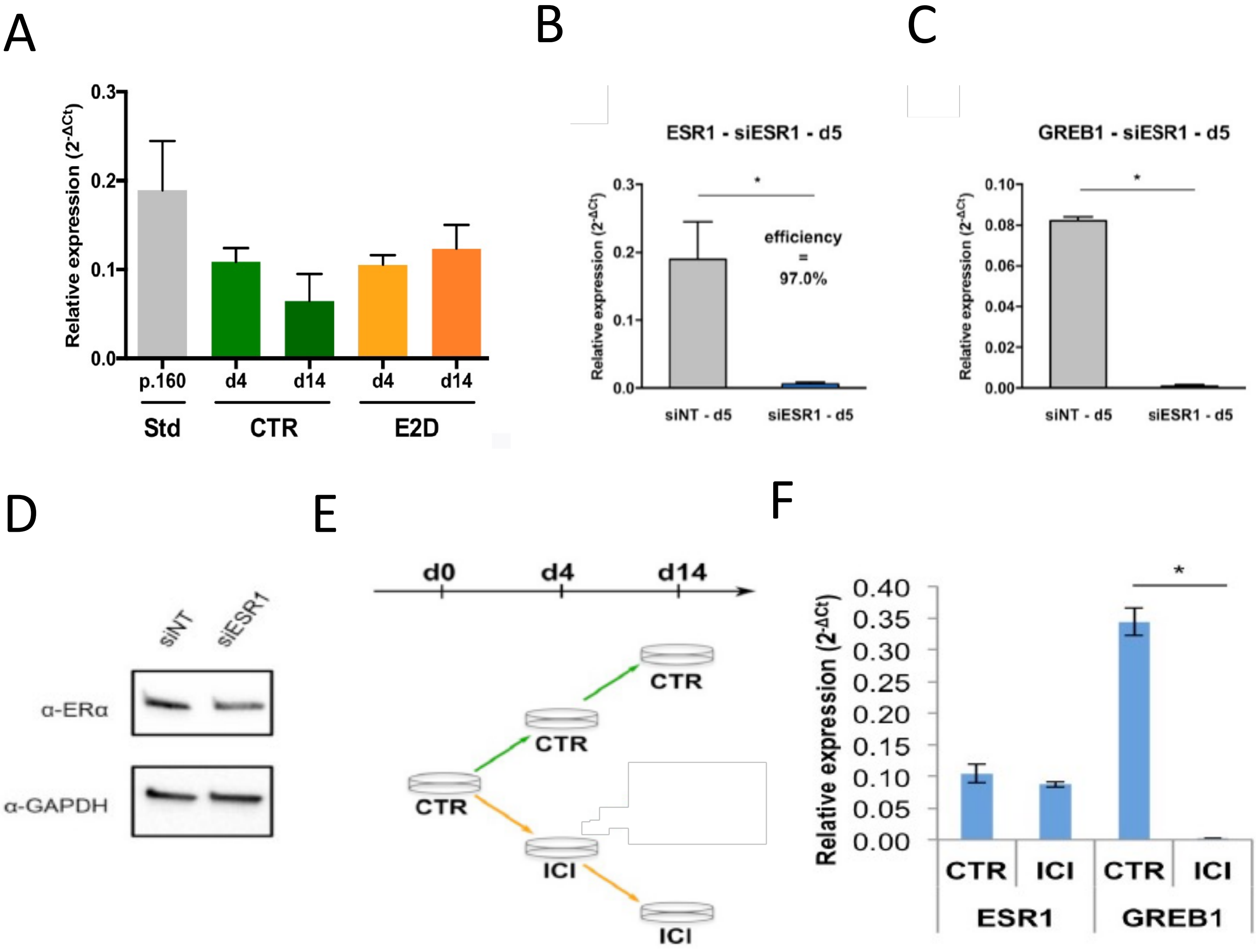

Supplementary Figure 1. **(A)** *ESR1* mRNA expression levelslevels in MCF-7 cells grown in standard medium till passage 160 (Std; phenol-red and complete FBS), in CTR and in E2D. GAPDH was used as an internal control. (n=3). **(B)** Relative expression of ESR1 5 days after transfection with a corresponding siRNA. **(C)** Effect of siESR1 on canonical ER target GREB1. Data shown as average ± SD of triplicates and was analysed using a t-test (* $p < 0.05$). **(D)** Protein levels of ESR1 after 5 days of transfection using a corresponding siRNA. GAPDH is shown for a positive control. **(E)** MCF-7 cells were treated with 0.1% DMSO (CTR, green) or 1µM of ICI 182,780 (ICI, orange dashed) for 4 and 14 days. (F) Relative expression of ESR1 and ER-target GREB1 after 4 days of ICI treatment

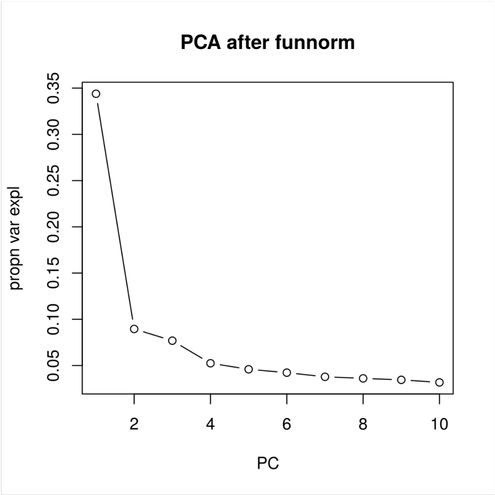

	PC1	PC2	PC3	PC4	PC5
**sentrix:**	0.1610.	477	0.144	**0.018**	0.526
**sentrix_position:**	0.297	0.576	0.752	0.931	0.243
**treatment:**	0.164	**0.037**	0.170	0.054	0.743
**time:**	0.395	**0.001**	0.661	0.166	0.288

Supplementary Figure 2. Treatment and time are the most significant variables that explain variability of DNAm in response to E2-deprivation after 14 days. Significance of principal components (pvalues) was evaluated with a Wilcoxon ranked test for two-factor variables (sentrix and treatment) and with a Kruskal-Wallis test for multi-factorial variables (sentrix-position and time).

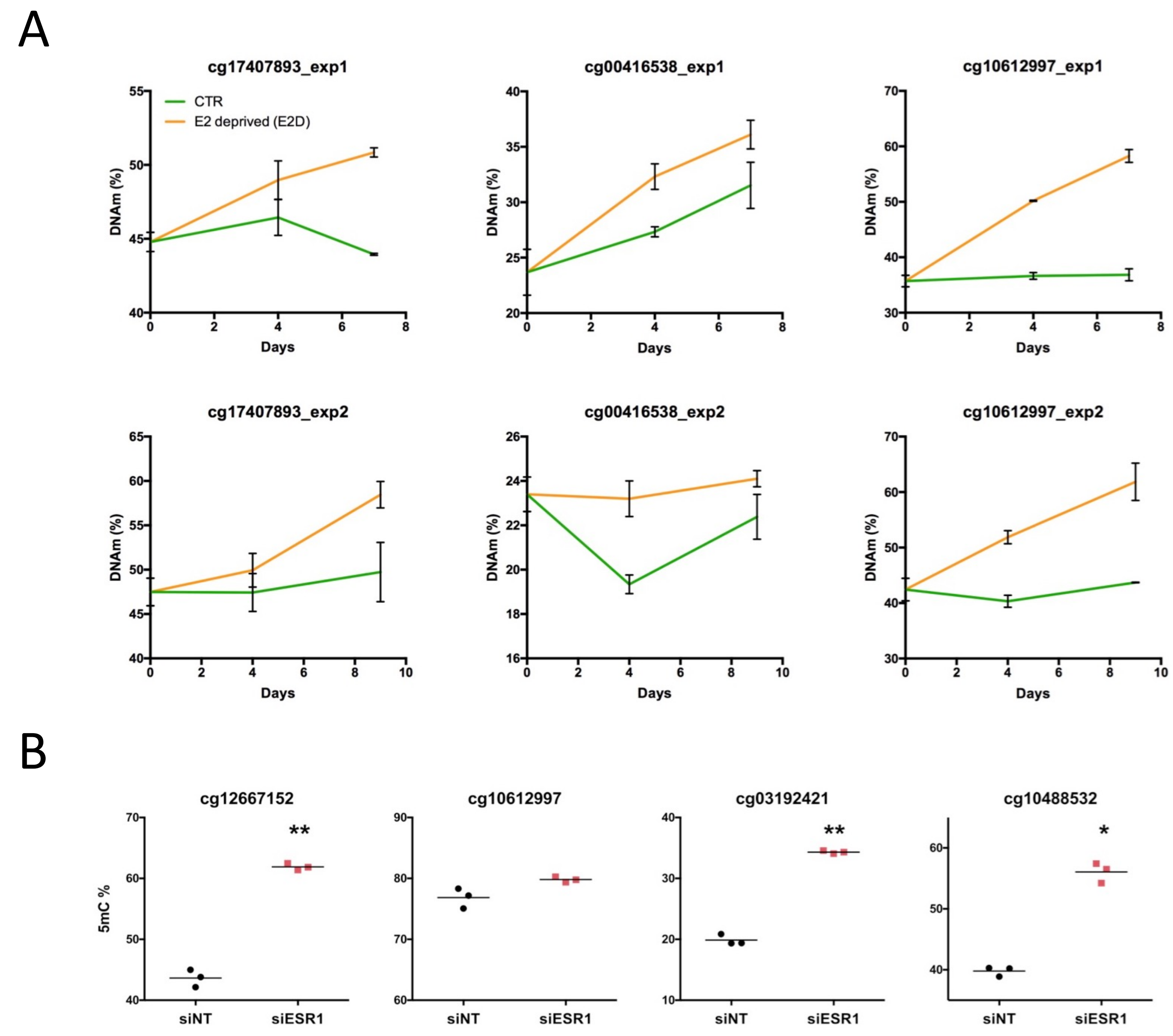

Supplementary Figure 3. **(A)** DNAm levels measured after 7 days (exp1) and 9 days (exp2) of E2 deprivation and **(B)** after 5 days of siRNA targeting ESR1 transcripts. DNAm was measured by pyrosequencing and is shown as average ± SD of triplicates. The effect of treatment was evaluated using a Mann-Whitney test.

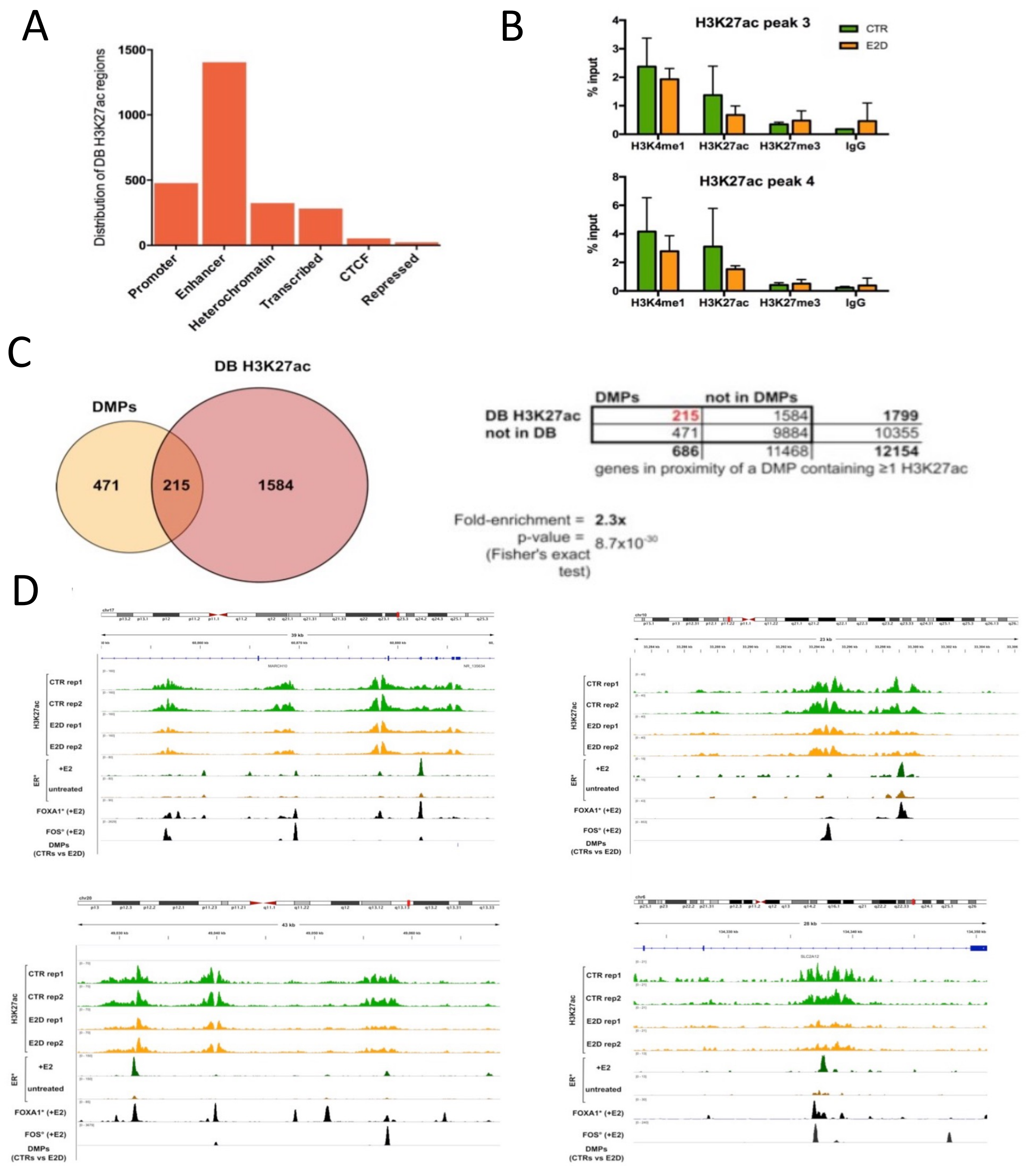

Supplementary Figure 4. **(A)** Overlap of DB H3K27ac regions with ChromHMM annotations coming from MCF-7 cells (Taberlay, 2014). **(B)** Validation of H3K27ac peaks as well as H3K4me1, H3K27me3 marks and IgG after 11 days of CTR and E2D treatments. qPCR data was normalized over input DNA and presented as the average ± SD of duplicates. Groups were compared using a Mann-Whitney test within each histone mark. **(C)** Pairwise overlap of genes that map nearby a DMP and a DB H3K27ac peak. Only genes present in both datasets were considered for fold-enrichment. **(D)** Genome browser snapshots of H3K27ac normalised ChIP-seq reads in CTR and E2D conditions, ER and FOXA1 ChIP-seq reads in E2-treated and untreated MCF-7 (*GSE72249, Swinstead et al., 2016) and FOS ChIP-seq (°GSE105734, ENCODE Project Consortium et al., 2012).

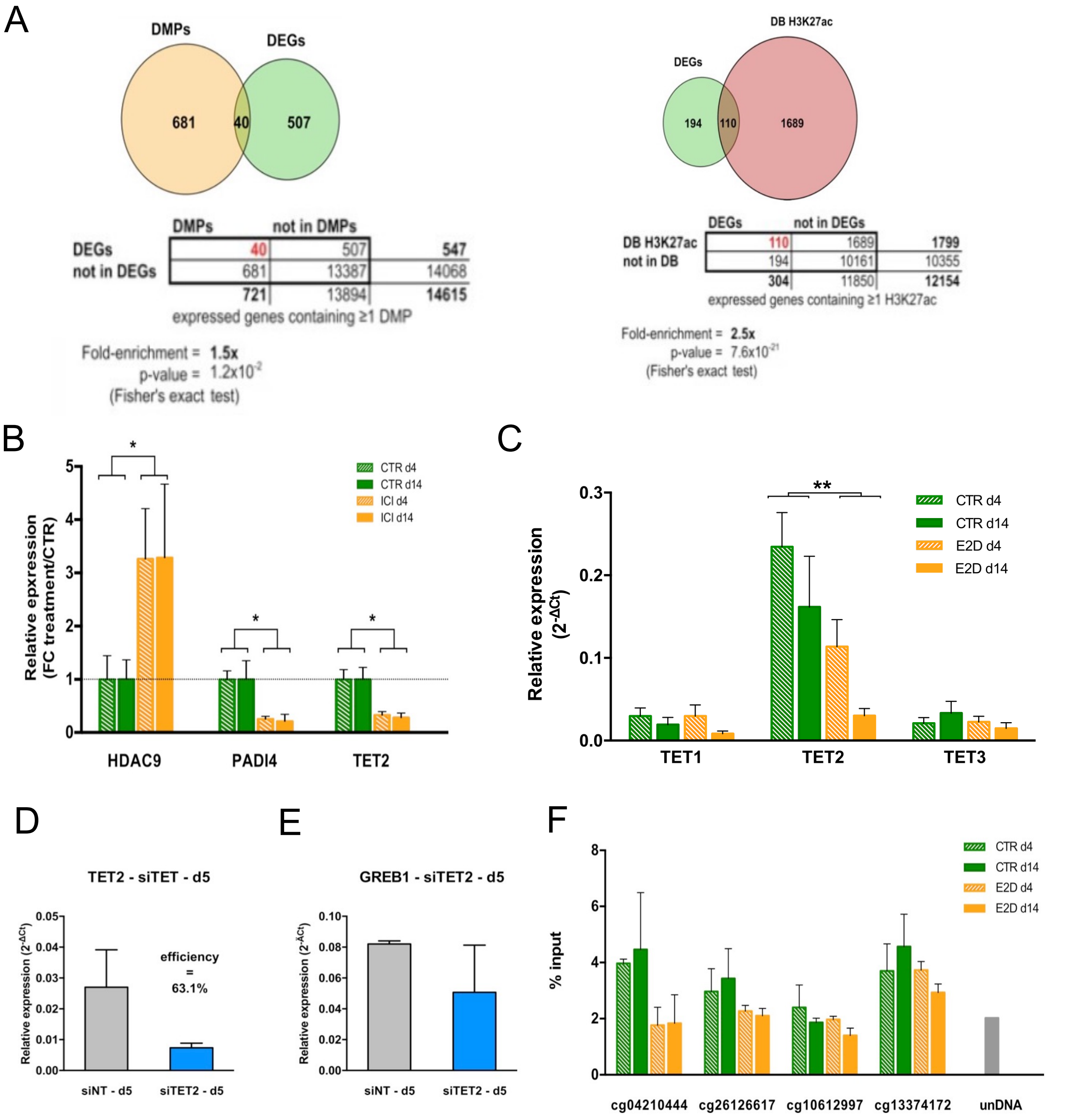

Supplementary Figure 5. **(A)** Pairwise overlap of DEGs and genes that map nearby a DMP (left) or a DB H3K27ac peak (right). Only genes present in both datasets were considered for fold-enrichment. **(B)** Expression of epigenetic remodeling factors HDAC9, PADI4 and TET2 in response to ER-inhibiting ICI treatment (RT-qPCR, Mann-Whitney CTR vs ICI, * p <0.01), for 4 and 14 days. Data of ICI treatment are shown as the average of triplicates ± SD. **(C)** Expression of TET1, TET2 and TET3 for 4 and 14 days E2 deprivation (RT-qPCR, Mann-Whitney CTR vs E2D, ** p <0.01). Data are shown as the average of triplicates ± SD. **(D)** Relative expression of TET2 5 days after transfection with a corresponding siRNA. **(E)** Effect of siTET2 KD on canonical ER target GREB1. Data shown as average ± SD of triplicates and was analysed using a t-test (* p <0.05). **(F)** Enrichment of 5-hydroxymethylation in CTR and E2D groups at top hyperDMP sites. Unmethylated DNA (unDNA) was spiked in one of the samples as a negative control. Data are shown as the average of biological triplicates ± SD.

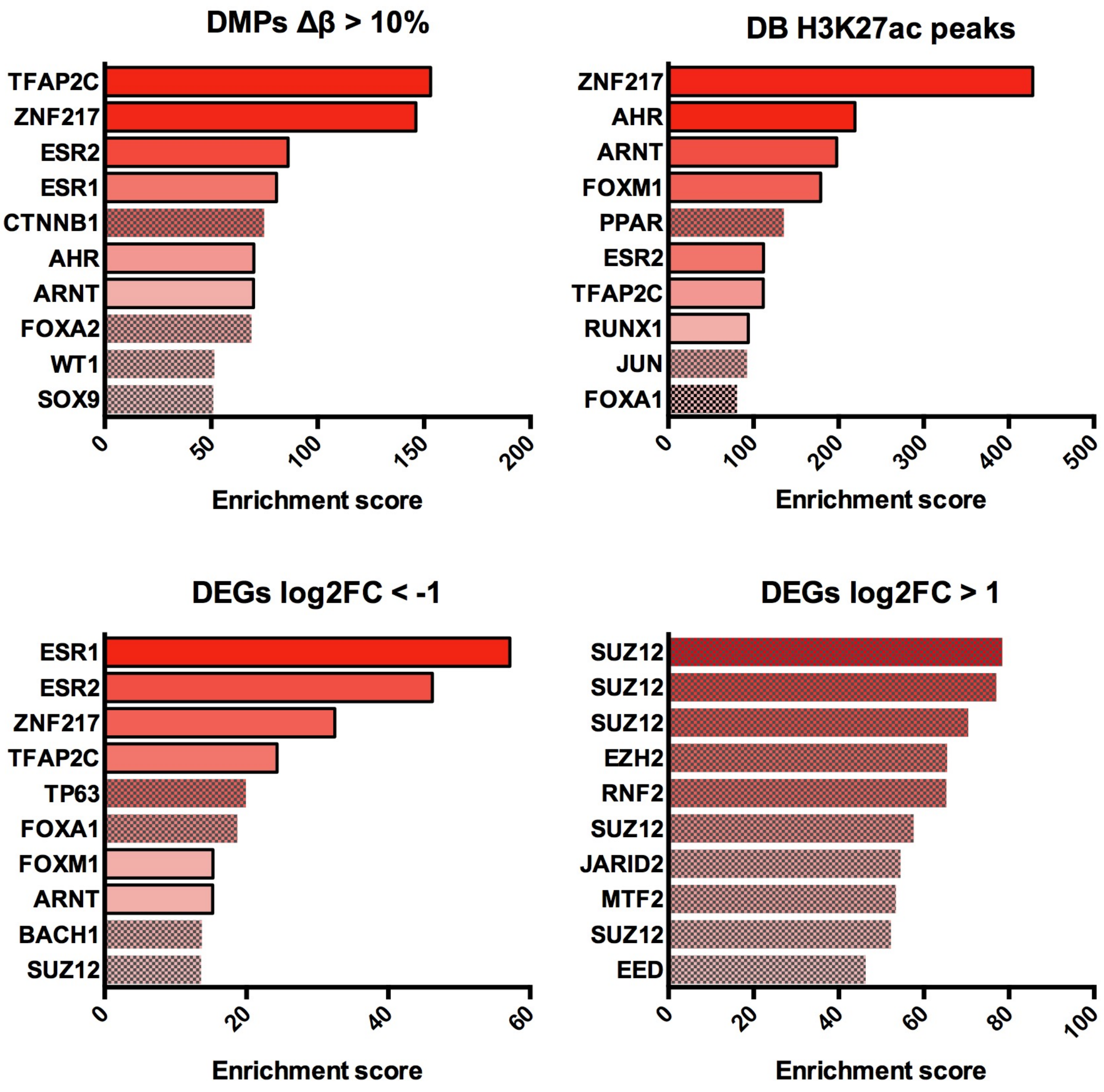

Supplementary Figure 6. Enrichment analysis of TF binding across the nearest genes to DMPs (Δβ > 10%, FDR <0.05, n=829), differentially acetylated histones (FDR <0.05, n=834) and down- and upregulated genes (logFC< -1 and 1, FDR < 0.05, n=381 and n)166)). Enrichment score is the combination of log(p-value) and Z-score of expected rank (ChEA 2016, EnrichR). Bars surrounded with a black line point out datasets that are originating from MCF-7 cells.

ChIP-seq ER (GSE72249, n=19858) (top 4 hits shown)

Motif	*p*-value	Enrichment (%observed /%backgr)	Best match (protein family)
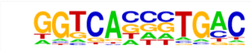	1E-1434	4.9x (24.9/5.1)	ER
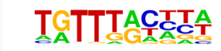	1E-1216	2.9x (36.0/12.3)	FOX
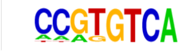	1E-1050	2.2x (46.5/21.2)	MEIS
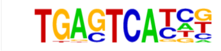	1E-420	2.8x (15.1/5.4)	AP-1

ChIP-seq FOS (GSE105734 n=63027) (top 4 hits shown)

Motif	*p*-value	Enrichment (%observed /%backgr)	Best match (protein family)
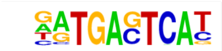	1E-28640	14.5x (66.8/4.5)	AP-1
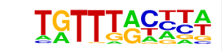	1E-725	1.8x (21.8/13.1)	FOX
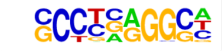	1E-264	1.6x (13.1/8.3)	AP-2γ
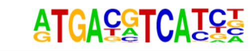	1E-165	2.5x (2.5/1.0)	ATF (CRE)

ChIP-seq FOXA1 (GSE72249 n=47893) (top 4 hits shown)

Motif	*p*-value	Enrichment (%observed /%backgr)	Best match (protein family)
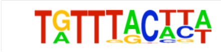	1E-4908	3.0x (50.9/16.9)	FOX
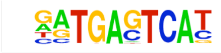	1E-1024	3.0x (13.6/4.6)	AP-1
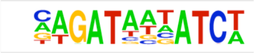	1E-604	3.2x (7.4/2.3)	GATA
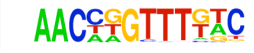	1E-376	1.7x (15.7/9.0)	GRHL

ChIP-seq TET2 (GSE153251 n=16842) (top 4 hits shown)

Motif	*p*-value	Enrichment (%observed /%backgr)	Best match (protein family)
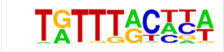	1E-1221	3.6x (31.2/8.7)	FOX
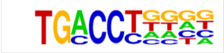	1E-1119	2.0x (56.7/27.5)	NR
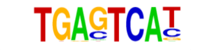	1E-474	2.9x (17.5/6.0)	AP-1
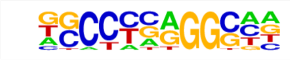	1E-291	1.3x (25.4/23.7)	AP-2γ

ChIP-seqp300 (GSE40129 n=23465) (top 4 hits shown)

Motif	*p*-value	Enrichment (%observed /%backgr)	Best match (protein family)
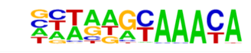	1E-1283	2.4x (41.7/17.5)	FOX
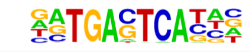	1E-1080	4.5x (17.3/3.9)	AP-1
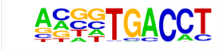	1E-719	1.7x (49.7/29.5)	NR
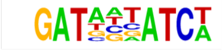	1E-345	3.2x (8.7/2.7)	GATA

ChIP-seq GATA3 (GSE127656 n=56436) (top 4 hits shown)

Motif	*p*-value	Enrichment (%observed /%backgr)	Best match (protein family)
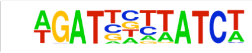	1E-2506	7.4x (11.2/1.5)	GATA
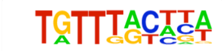	1E-1309	.9x (31.2/16.1)	FOX
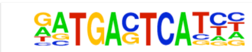	1E-1219	3.1x (13.0/4.1)	AP-1
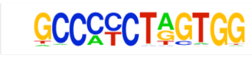	1E-1014	4.6x (7.0/1.5)	CTCF/Zic

Supplementary Figure 7. *De novo* motifs found in regions bound by ER and known cofactors.

A

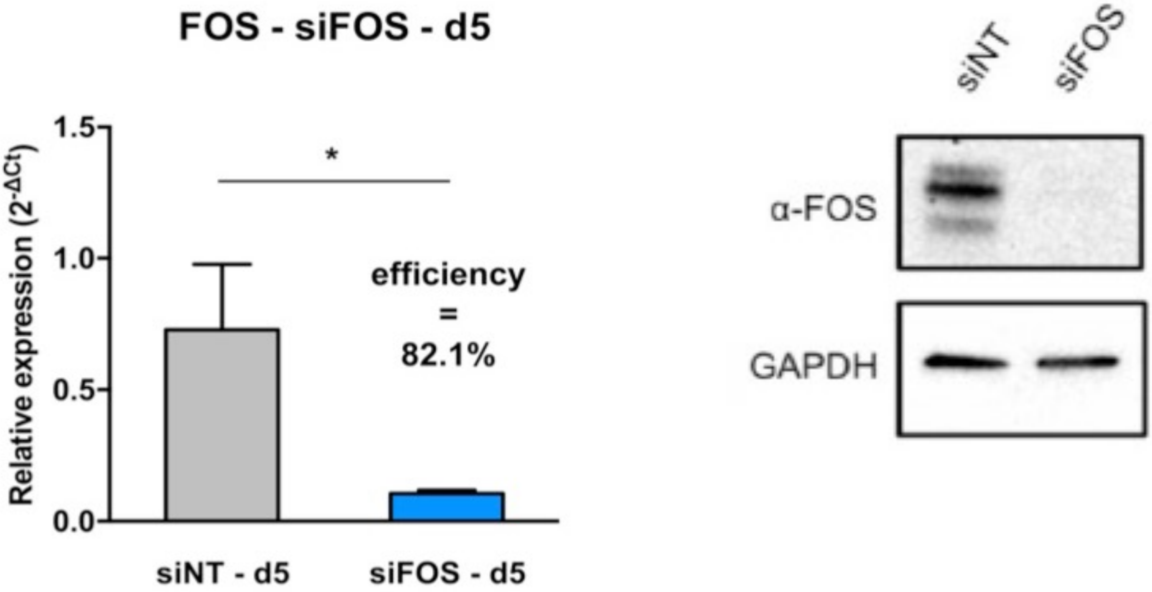

B

JUN binding	MCF-7	Tamoxifen-resistant MCF-7
DMPs	2	116
DB H3K27ac (loss)	124	792
DB H3K27ac (gain)	1	6

Supplementary Figure 8. **(A)** Relative expression of FOS (left) and protein levels of FOS (right) after 5 days of transfection using a corresponding siRNA. GAPDH is shown for a positive control. **(B)** Overlap of JUN bound regions detected in MCF-7 and tamoxifen-resistant MCF-7 cells (detected by Bi *et al* 2020) with DMPs and DB H3K27ac peaks (detected in E2D cells vs CTR).

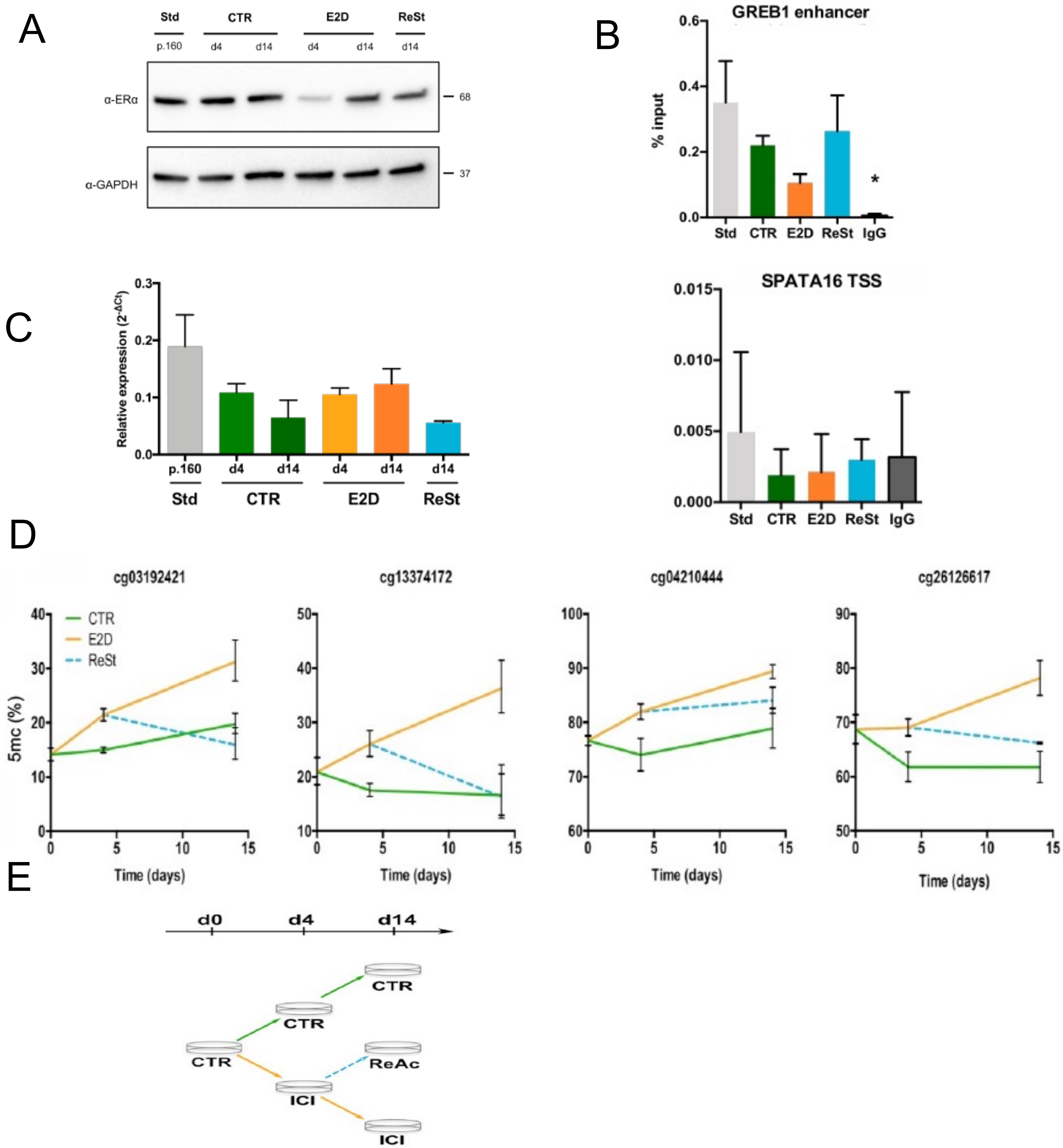

Supplementary Figure 9. **(A)** ERα (ER) protein levels in MCF-7 cells grown in standard medium till passage 160 (Std; phenol-red and complete FBS), in CTR, in E2D and in ReSt conditions. GAPDH was used as an internal control. **(B)** ER binding levels in GREB1 enhancer (up) and in SPATA16 promoter (down) for the same conditions as in (A) at d14 alone, (n=3). Asterisks indicate significant differences from the IgG (Student's t-test, $p< 0.05$). n.s., not significant (Student's t-test, $p> 0.05$) **(C)** *ESR1* mRNA expression levels for the same conditions as in (A) (n=3). **(D)** Technical validation of DNAm levels at top DMPs in CTR, E2D and ReSt (green, orange and blue) by pyrosequencing. **(E)** MCF-7 cells were treated with 0.1% DMSO for 4 and 14 days (CTR, green), 1μM ICI 182,780 for 4 and 14 days (ICI, orange) and 1μM of ICI 182,780 for 4 days, followed of 10 days without the inhibitor (ReAc, blue).

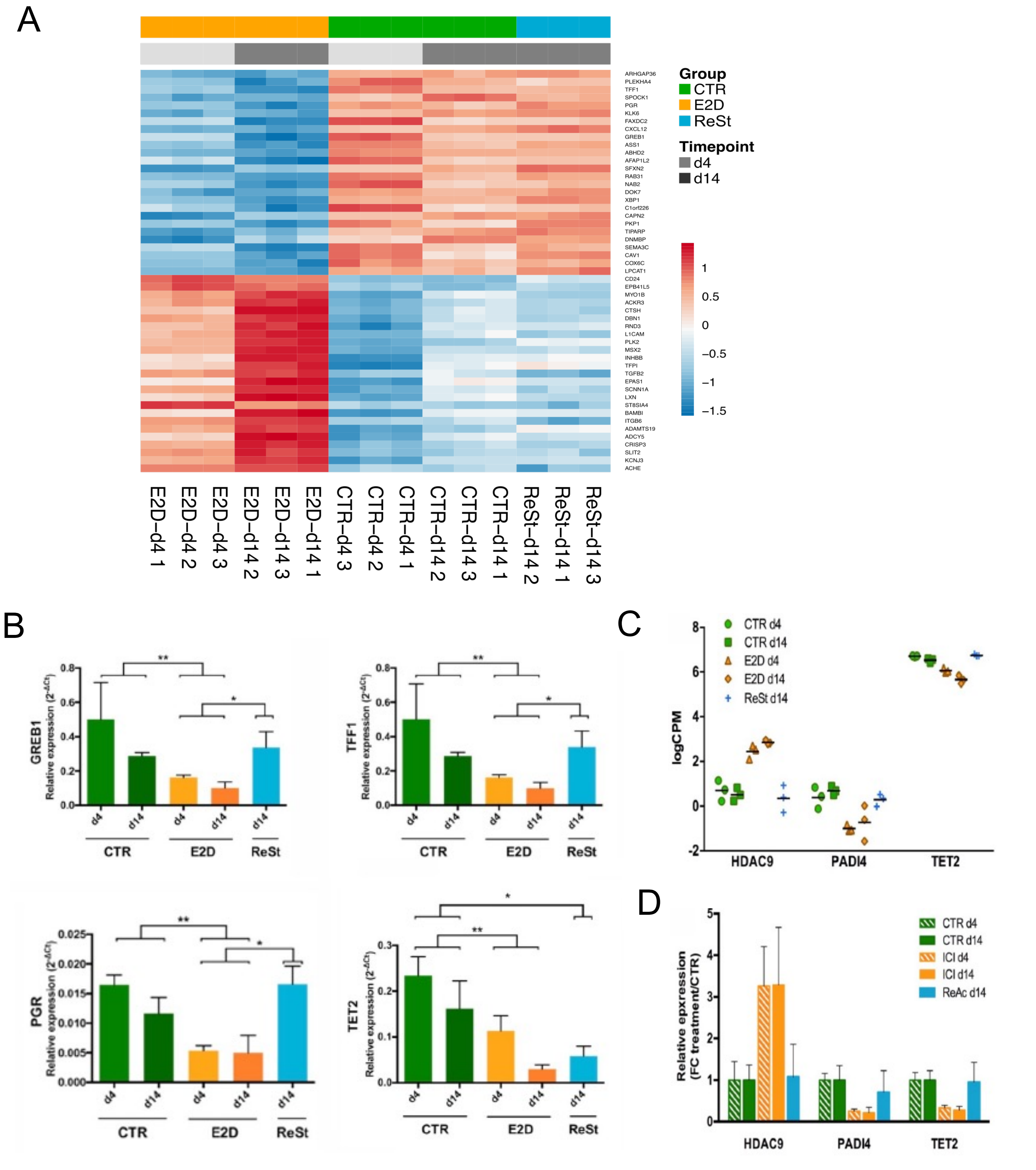

Supplementary Figure 10. **(A)** Top 50 differentially expressed genes (FDR<0.05) between CTR, E2D and ReSt. **(B)** Technical validation of ER targets and TET2 by RT-qPCR. The effect of treatment was tested using a Mann-Whitney test (* p <0.05; ** p <0.01). Results are shown as the average of triplicates ± SD. **(C)** Expression of epigenetic remodeling factors HDAC9, PADI4 and TET2 in response to E2 deprivation and re-stimulation (RNA-seq, FDR < 0.05). **(D)** Expression of epigenetic remodeling factors HDAC9, PADI4 and TET2 in response to ER-inhibiting ICI treatment (RT-qPCR), for 4 and 14 days and to 4 days of ICI followed by 10 days of re-activation (ReAc). Data of ICI treatment are shown as the average of triplicates ± SD.

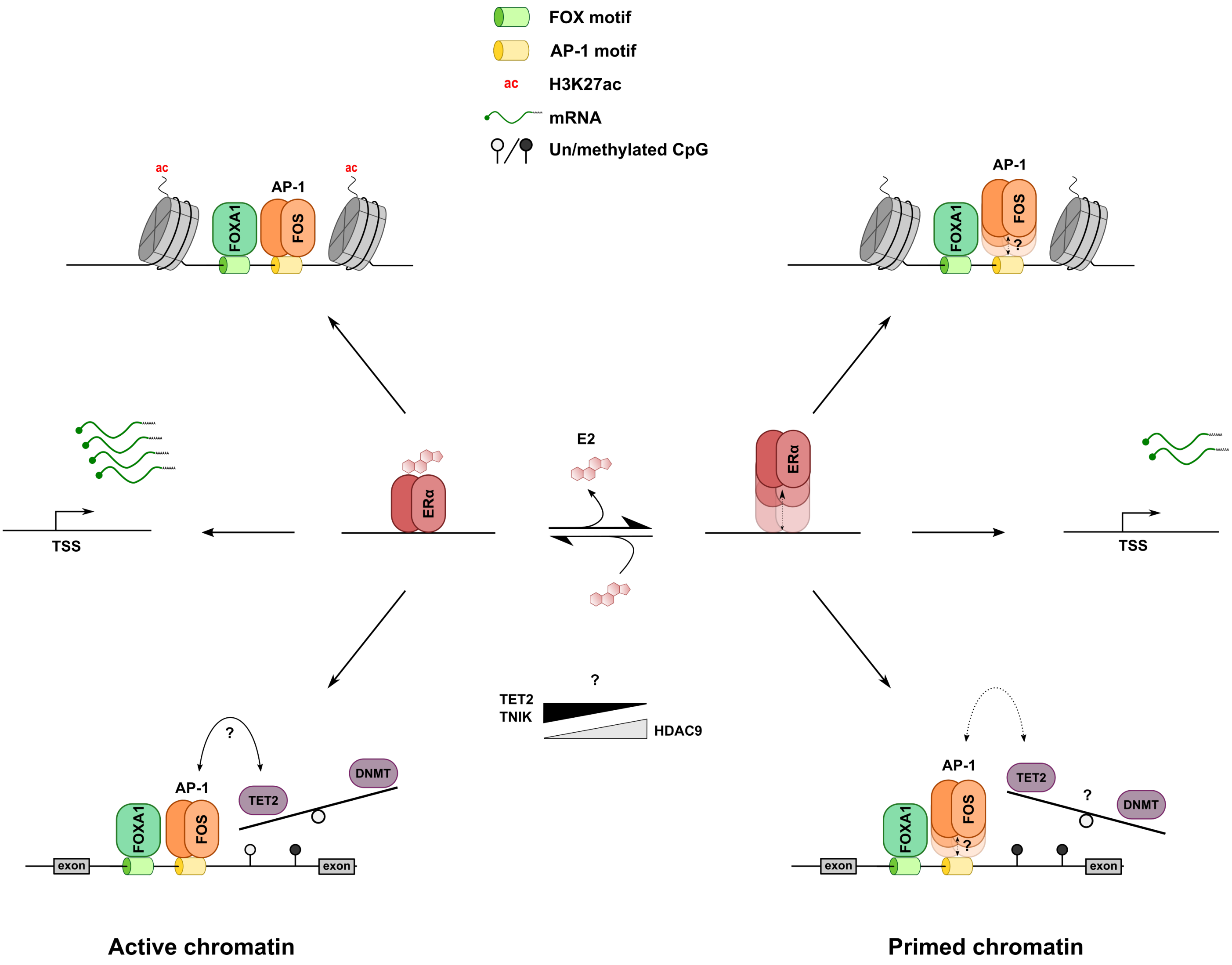

Supplementary Figure 11. Shift from active to primed chromatin state in response to E2 deprivation and re-stimulation. Mechanistic model by which the reduction of ligand-dependent ER activity leads to an AP-1 and FOXA1 mediated increase of DNAm and decrease of H3K27ac and transcription. The reversibility of these changes is partial. This model integrates current findings to prior knowledge of ER-dependent chromatin and TF interactions.

Supplementary table 1. Primer sequences to measure DNA methylation by pyrosequencing. Primers carrying a BIO at their 5' contain "BIO" in their name

Primer	Sequence	Sequencing primer	Genomic range	Amplicon (bp)	Annealing Temp (°C)
cg12667152_F<br>cg12667152_BIO_R	AGTTATGATGGGGTGGAGAAA<br>AAAATCCCTACTAATCCACACAAA	Same as forward	chr11:85,884,137-85,884,246	110	<55.8
cg10612997_F<br>cg10612997_BIO_R	TGTGTTATTGATGAAGTTTTTGAG<br>ACTAAAATCTACACACCATACTCTCC	TTAGGGGAGAATATG	chr2:11,673,902-11,674,119	218	61
cg03192421_F<br>cg03192421_BIO_R	AGGATTGTGAATATTATGTTATTGTG<br>CAAACCAAAATCAACCTCAA	GTTTATAATGGTAAT	chr20:10,643,103-10,643,295	193	56
cg04210444_F<br>cg04210444_BIO_R	GTTTGTGTGAATAGAGGTTTTAGG<br>CACACTACCTTCTCTTATCAATTATCA	Same as forward	chr20:46,397,276-46,397,410	135	56
cg17407893_BIO_F<br>cg17407893_R	GTGGTATTTGGGAATTTGTTG<br>CAAAAACCCTCCCACTAAATT	GTAATTTGTGTTTTA	chr16:16,119,175-16,119,303	129	61
cg13670878_BIO_F<br>cg13670878_R	AGTGGATGGTAAGTGAGAGG<br>CAACCTAAACCCTACTAAAATATTTA	TATTTAAAATTAAC	chr2:11,681,555-11,681,896	342	58
cg13374172_F<br>cg13374172_BIO_R	TATGTTTGAGTTAGTAAGAGGGG<br>CCCATACATAATACTTAAAATATCACTT	Same as forward	chr8:56,860,886-56,861,092	207	56
cg26126617_F<br>cg26126617_BIO_R	GGTTTGGATTAAATAGAGGAGTATTTG<br>CCAAAACCCCTAAATAATATAACCAC	Same as forward	chr2:25,077,082-25,077,220	139	56

Supplementary table 2. Primer sequences to measure histone mark enrichment by qPCR

Primer	Sequence	Amplicon (bp)	Source
Peak1_F<br>Peak1_R	GCTTGCAGGCAGAAATGTAAACAC<br>CTGTTGCCCTAAGCCTGTATT	136	designed in this study
Peak2_F<br>Peak2_R	GTTGTGTGGACCATATTTTCTAAAC<br>TGTTTACATTTCTGCCTGCA	102	designed in this study
Peak3_F<br>Peak3_R	TTAAACAGTTCTCCTCCACTCC<br>GCAGATTACAAAACTCACTGAAGAC	74	designed in this study
Peak4_F<br>Peak4_R	TGATGTGTCAGGAACCCTCC<br>CCCAGACCTACTGCATCAGAAAC	92	designed in this study
GREB1_pos_F<br>GREB1_pos_R	CACGTCCCCACCTCACTG<br>TGTTCAGCTTCGGGACACC	56	Jozwik, 2016, Cell Reports
SPATA16_neg_F<br>SPATA16_neg_R	GACGCCAGGACTTGAAAC<br>TCAAGTGCAACTCAAGAGGA	102	designed in this study

Supplementary table 3. Primer sequences to measure gene expression

Primer	Sequence	Amplicon (bp)	Source
qRT_GREB1_F<br>qRT_GREB1_R	ATGGGAAATTCTTACGCTGGAC<br>CACTCGGCTACCACCTTCT	171	designed in this study
qRT_TFF1_F<br>qRT_TFF1_R	TCGAAACAGCAGCCCTTATT<br>GGCCCAGACAGAGACGTGTA	102	designed in this study
qRT_PGR_F<br>qRT_PGR_R	TTATGGTGTCCTTACCTGTGGG<br>GCGGATTTTATCAACGATGCAG	112	designed in this study
qRT_FOS_F<br>qRT_FOS_R	AAGCGGAGACAGACCAACTA<br>CAGGTCATCAGGGATCTTG	134	designed in this study
qRT_JUN_F<br>qRT_JUN_R	AACAGGTGGCACAGCTTAAAC<br>CAACTGCTGCGTTAGCATGAG	77	PrimerBank ID 44890066c2
qRT_HDAC9_F<br>qRT_HDAC9_R	AGTAGAGAGGCATCGCAGAGA<br>GGAGTGTCTTTCGTTGCTGAT	141	PrimerBank 116284378c1
qRT_PADI4_F<br>qRT_PADI4_R	CAGGGGACATTGATCCGTGTG<br>GGGAGGCGTTGATGCTGAA	130	PrimerBank 216548486c1
qRT_MMP13_F<br>qRT_MMP13_R	ATTTCTCGGAGCCTCTCAGT<br>AGTTTGCAGAGCGCTACCT	103	designed in this study
qRT_RPLP0_F<br>qRT_RPLP0_R	TGGCAGCATCTACAACCCTGAA<br>ACACTGGCAACATTGCGGACA	90	designed in this study
qRT_TNIK_F<br>qRT_TNIK_R	TAAGGGTCGTCATGTCAAAACG<br>CCATGCCTGGTGGGTTCTTT	173	PrimerBank 239735578c1
qRT_TET2_F<br>qRT_TET2_R	CTTTCCTCCCTGGAGAACAGCTC<br>TGCTGGGACTGCTGCATGACT	146	designed in this study

Supplementary table 4. List of published ChIP-seq datasets produced in MCF-7

GEO series	Target	Format	Year	Reference
GSE75070	RUNX1	bed	2016	Barutcu AR, Hong D, Lajoie BR, McCord RP et al. RUNX1 contributes to higher-order chromatin organization and gene regulation in breast cancer cells. Biochim Biophys Acta 2016 Nov;1859 (11):1389-1397. PMID: 27514584
GSE96352	H3K27ac	bed	2017	ENCODE Project Consortium. An integrated encyclopedia of DNA elements in the human genome. Nature 2012 Sep 6;489 (7414):57-74. PMID: 22955616
GSE96363	H3K27me3	bed	2017	ENCODE Project Consortium. An integrated encyclopedia of DNA elements in the human genome. Nature 2012 Sep 6;489 (7414):57-74. PMID: 22955616
GSE31477	ZNF217	bed	2012	ENCODE Project Consortium. An integrated encyclopedia of DNA elements in the human genome. Nature 2012 Sep 6;489 (7414):57-74. PMID: 22955616
GSE105734	FOS	bed; bw	2017	ENCODE Project Consortium. An integrated encyclopedia of DNA elements in the human genome. Nature 2012 Sep 6;489 (7414):57-74. PMID: 22955616
GSE91550	JUN	bed	2017	ENCODE Project Consortium. An integrated encyclopedia of DNA elements in the human genome. Nature 2012 Sep 6;489 (7414):57-74. PMID: 22955616
GSE32465	JUND	bed	2017	ENCODE Project Consortium. An integrated encyclopedia of DNA elements in the human genome. Nature 2012 Sep 6;489 (7414):57-74. PMID: 22955616
GSE127656	GATA3	bed	2016	ENCODE Project Consortium. An integrated encyclopedia of DNA elements in the human genome. Nature 2012 Sep 6;489 (7414):57-74. PMID: 22955616
GSE106068	CREB1	bed	2017	ENCODE Project Consortium. An integrated encyclopedia of DNA elements in the human genome. Nature 2012 Sep 6;489 (7414):57-74. PMID: 22955616
GSE91415	CUX1	bed	2017	ENCODE Project Consortium. An integrated encyclopedia of DNA elements in the human genome. Nature 2012 Sep 6;489 (7414):57-74. PMID: 22955616
GSE106025	ATF-7	bed	2017	ENCODE Project Consortium. An integrated encyclopedia of DNA elements in the human genome. Nature 2012 Sep 6;489 (7414):57-74. PMID: 22955616
GSE105740	NCOA3	bed	2017	ENCODE Project Consortium. An integrated encyclopedia of DNA elements in the human genome. Nature 2012 Sep 6;489 (7414):57-74. PMID: 22955616
GSE32465	HDAC2	bed	2012	ENCODE Project Consortium. An integrated encyclopedia of DNA elements in the human genome. Nature 2012 Sep 6;489 (7414):57-74. PMID: 22955616
GSE32465	FOSL2	bed	2012	ENCODE Project Consortium. An integrated encyclopedia of DNA elements in the human genome. Nature 2012 Sep 6;489 (7414):57-74. PMID: 22955616
GSE85106	CTCF	bed	2016	Fiorito E, Sharma Y, Gilfillan S, Wang S et al. CTCF modulates Estrogen Receptor function through specific chromatin and nuclear matrix interactions. Nucleic Acids Res 2016 Dec 15;44 (22):10588-10602. PMID: 27638884
GSE81714	H3K4me1	bed	2016	Jozwik KM, Chernukhin I, Serandour AA, Nagarajan S et al. FOXA1 Directs H3K4 Monomethylation at Enhancers via Recruitment of the Methyltransferase MLL3. Cell Rep (10):2715-2723. PMID: 279268732016 Dec 6;17
GSE81714	H3K4me3	bed	2016	Jozwik KM, Chernukhin I, Serandour AA, Nagarajan S et al. FOXA1 Directs H3K4 Monomethylation at Enhancers via Recruitment of the Methyltransferase MLL3. Cell Rep (10):2715-2723. PMID: 279268732016 Dec 6;17
GSE40767	FOXM1	bed	2013	Sanders DA, Ross-Innes CS, Beraldi D, Carroll JS et al. Genome-wide mapping of FOXM1 binding reveals co-binding with estrogen receptor alpha in breast cancer cells. (1):R6. PMID: 23347430 Genome Biol 2013 Jan 24;14
GSE71327	RACK7	bed	2016	Shen H, Xu W, Guo R, Rong B et al. Suppression of Enhancer Overactivation by a RACK7-Histone Demethylase Complex. Cell 2016 Apr 7;165 (2):331-42. PMID: 27058665

GSE72249	FOXA1	bed; bw	2016	Swinstead EE, Miranda TB, Paakinaho V, Baek S et al. Steroid Receptors Reprogram FoxA1 Occupancy through Dynamic Chromatin Transitions. Cell 2016 Apr 21;165 (3):593-605. PMID: 27062924
GSE72249	ER	bed; bw	2016	Swinstead EE, Miranda TB, Paakinaho V, Baek S et al. Steroid Receptors Reprogram FoxA1 Occupancy through Dynamic Chromatin Transitions. Cell 2016 Apr 21;165 (3):593-605. PMID: 27062924
GSE23852	TFAP2C (AP-2γ)	bed	2011	Tan SK, Lin ZH, Chang CW, Varang V et al. AP-2γ regulates oestrogen receptor-mediated long-range chromatin interaction and gene transcription. EMBO J 2011 May 13;30 (13):2569-81. PMID: 21572391
GSE40129	p300	bed	2012	Theodorou V, Stark R, Menon S, Carroll JS. GATA3 acts upstream of FOXA1 in mediating ESR1 binding by shaping enhancer accessibility. Genome Res 2013 Jan;23 (1):12-22. PMID: 23172872
GSE90550	AHR	bed	2018	Yang SY, Ahmed S, Satheesh SV, Matthews J. Genome-wide mapping and analysis of aryl hydrocarbon receptor (AHR)- and aryl hydrocarbon receptor repressor (AHRR)-binding sites in human breast cancer cells. Arch Toxicol 2018 Jan;92 (1):225-240. PMID: 28681081
GSE128460	JUN	bed bw	2020	Mingjun Bi, Zhao Zhang, Yi-Zhou Jiang, Pengya Xue, Hu Wang, Zhao Lai, Xiaoyong Fu, Carmine De Angelis, Yue Gong, Zhen Gao, Jianhua Ruan, Victor X. Jin, Elisabetta Marangoni, Elodie Montaudon, Christopher K. Glass, Wei Li, Tim Hui-Ming Huang, Zhi-Ming Shao, Rachel Schiff, Lizhen Chen & Zhijie Liu. Enhancer reprogramming driven by high-order assemblies of transcription factors promotes phenotypic plasticity and breast cancer endocrine resistance. Nature Cell Biology volume 22, pages701–715 (2020). PMID: 32424275

Supplementary table 5. ER, AP-1 and FOXA-1 motif occurrence within ER binding sites (n=19858, ChIP-seq GSE72249 (Swinstead et al 2016)

Genomic feature	Count of ER motif	Count of AP-1 motif	Count of FOXA1 motif
3' UTR	120	112	213
5' UTR	69	55	86
Distal Intergenic	2598	3887	6613
Downstream	52	70	96
Exon	261	289	452
Intron	2446	3336	5725
Promoter	240	259	347
**Grand Total**	**5786**	**8008**	**13532**
